# A Study on the Mechanism of N-Carbamylglutamate’s (NCG) Effects on the Liver Transcriptome and Metabolism of Cold-Stressed Mongolian Ewes and Fetuses

**DOI:** 10.3390/ani16182932

**Published:** 2026-09-18

**Authors:** Kang Li, Yu Zhi, Lulu Shi, Jing Tian, Yunhua Li, Juan Du, Meimei Zhang, Hao Zhang, Zhong Dong, Ruijun Wang, Weiyong Guo, Tianlong Guo

**Affiliations:** 1 Inner Mongolia Academy of Agricultural & Animal Husbandry Sciences, Hohhot 010030, China; 13947110359@163.com (K.L.); zhiyu6836@163.com (Y.Z.); 15024913949@163.com (L.S.); tianjing_0915@126.com (J.T.); yhli5277@163.com (Y.L.); dujuan_ddj@163.com (J.D.); 2 Jungar Banner Bureau of Agriculture and Animal Husbandry, Ordos 017000, China; zmm5641@163.com; 3 Inner Mongolia Chia Tai Co., Ltd., Hohhot 010030, China; 15184720862@163.com (H.Z.); dongzhong@126.com (Z.D.); wangrh19820301@163.com (R.W.); 4 Shan County Animal Husbandry Service Center, Heze 274300, China; sxdj75@163.com

**Keywords:** N-carbamylglutamate, cold-stress, Mongolia sheep, metabolism regulation, late gestation

## Abstract

Chronic cold stress disrupts hepatic metabolism and fetal development of Mongolian sheep in northern pastoral zones, while systematic nutritional mitigation strategies remain poorly characterized. N-carbamylglutamate (NCG) promotes endogenous arginine synthesis; however, its hepatic regulatory roles against cold stress in pregnant ewes and fetuses remain unclear. This study explored the potential protective effects of NCG via integrated hepatic transcriptome and metabolome analysis. Twenty-four late-gestation ewes were assigned to warm control, cold stress, and cold stress + NCG supplementation groups. Hepatic mRNA, lncRNA, circRNA, and miRNA sequencing, untargeted LC-MS metabolomics, multi-omics conjoint analysis, and qRT-PCR verification were performed using ewe and fetal liver tissues. The results indicated that cold stress potentially was associated with mTOR, PI3K/AKT, amino acid, and energy metabolic pathways, inducing extensive transcriptomic and metabolic alterations. NCG supplementation might reverse cold-induced transcriptional and metabolic disorders and restore glutathione and cysteine–methionine metabolism. qRT-PCR validated the differential expression of core hub genes AK4, AHCY, and RRM2, linking transcriptomic and metabolic reprogramming. Divergent hepatic molecular responses to NCG were observed between ewes and fetuses. Multi-omics correlation analysis suggested that maternal NCG may modulate hepatic energy and amino acid metabolism through mTOR and redox-related pathways under cold stress and potentially exert transgenerational hepatic effects on fetuses. These findings provide preliminary omics-based mechanistic hypotheses and a theoretical basis for nutritional intervention against cold stress in sheep production.

## Simple Summary

Mongolian sheep are raised in the cold regions of northern China, and winter cold imposes long-term cold stress on the flock: pregnant ewes consume extra energy to maintain body temperature, which disturbs normal liver function and often leads to weak lambs at birth, all of which cuts into farmers’ profits. We tested a natural feed additive that boosts a key nutrient, aiming to relieve cold stress and protect both ewes and their fetuses. Pregnant ewes were divided into three groups: one kept in a warm shelter, one exposed to outdoor cold, and one exposed to cold but given the additive every day. Examination of liver tissues showed that cold exposure disrupted normal gene activity and metabolic pathways, while the additive corrected most of these disturbances, helped restore balanced energy use, and protected the fetuses and ewes. This research offers farmers a practical feeding strategy to reduce cold-related losses, improve sheep health and lamb growth, and support sustainable sheep production on the northern grasslands.

## 1. Introduction

In livestock production, stress commonly encompasses thermal stress, transport stress, and weaning stress, with temperature fluctuations—cold and heat stress—having the most significant and widespread impacts on livestock. In northern regions, cold stress is particularly prevalent. Cold stress can be categorized into acute cold stress and chronic cold stress, depending on the duration of exposure to low temperatures [1]. Cold stress is a critical environmental factor limiting meat sheep production in northern pastoral areas and poses a persistent threat to the farming efficiency of local breeds such as the Mongolian sheep. Studies have indicated that cold stress not only increases energy expenditure and reduces feed utilization efficiency but also alters rumen fermentation, nutrient metabolism, and endocrine balance, thereby impacting nutrient allocation and fetal development during pregnancy [2].

Accumulating studies have revealed the physiological mechanisms of cold stress in ruminants, which severely impairs livestock growth, metabolism, reproduction and health. In sheep, cold stress retards growth and reduces feed conversion efficiency, damages wool development and reproductive performance, and increases lamb mortality, resulting in severe economic losses in animal husbandry. Metabolically, cold stress remodels rumen and cecal microbial communities, disrupts short-chain fatty acid metabolism and nutrient absorption [3], and reprograms carbohydrate and amino acid metabolism to elevate host energy expenditure. At the molecular level, it regulates hepatic energy metabolism pathways (e.g., mTOR signaling) and breaks rumen microbial homeostasis, thereby inducing ruminant metabolic disorders [4]. Cold stress also triggers endocrine and immune disorders [5], upregulates inflammatory factors such as IL-1β, and increases the risk of intestinal barrier damage [6] and metabolic disorders. In addition, cold stress impairs male reproductive performance via inhibiting sperm protease activity and disturbing cellular energy metabolism [7], and still compromises the vitality of newborn lambs [8] even after long-term genetic selection [9]. Moreover, it disrupts the oxidative balance of sheep by increasing xanthine oxidase activity and decreasing catalase activity, which causes excessive free radical accumulation, aggravated lipid peroxidation and eventual cellular oxidative damage [10]. Malondialdehyde (MDA) is a critical marker of oxidative injury, while T-SOD and CuZn-SOD serve as core antioxidant enzymes for free radical elimination [11]. However, current research primarily focuses on the negative impacts of cold stress and its physiological mechanisms, with relatively limited studies on nutritional interventions to alleviate cold stress and improve the metabolic states of ewes and their offspring.

N-carbamylglutamate (NCG), an endogenous N-acetylglutamate analog and arginine synthesis activator, targets the mitochondrial urea cycle to activate carbamoyl phosphate synthetase-I (CPS-I). It facilitates the synthesis of citrulline and endogenous arginine, thereby modulating nitrogen metabolism, protein synthesis and energy metabolism in vivo [12]. Existing studies have verified the beneficial functions of NCG in multiple animal models. In monogastric livestock, NCG improves growth and reproductive performance, reduces embryo loss and increases fetal birth weight, with its biological effects closely associated with AMPK, cGMP and mTOR signaling pathways [13]. For ruminants, dietary NCG supplementation can enhance the carcass performance of grazing sheep [14]. Moreover, NCG exerts crucial regulatory effects on maternal–fetal nutritional metabolism, oxidative stress resistance and intrauterine development. Maternal NCG supplementation optimizes fetal endocrine and metabolic homeostasis, elevates amino acid availability in fetal tissues, modulates growth hormone axis-related gene expression, and alleviates intrauterine growth restriction (IUGR) [15]. Studies have consistently demonstrated that NCG or rumen-protected arginine (RP-Arg) supplementation for malnourished pregnant ewes improves maternal endocrine status, enhances maternal-placental antioxidant capacity [16], and promotes placental development and amino acid transport throughout gestation [17]. Such nutritional interventions also attenuate IUGR-induced impairments in fetal thymic development and immune function, benefiting offspring immune potential [18]. A previous nutritional restriction model in pregnant Hu sheep further confirmed that NCG and RP-Arg regulate complex amino acid, carbohydrate, lipid and oxidative stress metabolic networks in fetal plasma, thereby improving maternal reproductive performance and fetal development [19]. Overall, NCG plays a positive role in improving maternal-placental–fetal nutritional metabolism, alleviating IUGR, and enhancing antioxidant capacity, yet its specific regulatory mechanisms under environmental stressors (such as cold stress) remain to be explored in greater depth. Despite these findings, systematic research on the potential of NCG to alleviate cold stress in ruminants, especially in grazing Mongolian sheep during harsh winter conditions, is still lacking.

Mongolian sheep, as a local breed well adapted to cold environments, exhibit unique resilience to cold stress and possess strong environmental adaptability and cold resistance, making them an ideal model for studying the mechanisms of cold adaptation in ruminants. The liver serves as the core organ for metabolic regulation, redox homeostasis, and immune response under stress conditions. The thermogenic response induced by low temperatures in liver tissue plays a crucial role in the survival of lambs exposed to cold [20].

Based on this, we propose the hypothesis that the inclusion of NCG in the diet can alleviate cold stress-induced metabolic disorders, oxidative stress, and inflammatory responses in the liver of pregnant ewes and their fetuses by regulating hepatic gene expression and metabolite profiles. The underlying mechanisms may involve the mTOR signaling pathway and oxidative stress-related pathways. To validate this hypothesis, we selected healthy pregnant Mongolian ewes and randomly divided them into three groups: a control group, a cold stress group, and a cold stress + NCG supplementation group. A chronic cold stress model was established, and NCG was supplemented periodically. We collected liver tissues from both ewes and fetuses for transcriptomic and metabolomic sequencing, followed by integrated analysis of the sequencing data to identify key differentially expressed genes and metabolites. Additionally, real-time quantitative PCR (qPCR) was employed to validate the differential genes, assessing the accuracy and reliability of the sequencing data. In summary, this study aims to clarify the molecular mechanisms through which NCG regulates hepatic transcription and metabolism in cold-stressed Mongolian ewes and fetuses via the mTOR signaling pathway and oxidative stress modulation, providing important theoretical and practical foundations for improving livestock health and production efficiency in cold environments, as well as supporting the development of nutritional strategies for stress management in ruminants.

## 2. Materials and Methods

### 2.1. Animals and Experimental Design

All animal procedures were in accord with and approved by the Animal Ethical Committee of Nanjing Agricultural University (SYXK2022-0031). This experiment was conducted in Siziwang Banner, Inner Mongolia, following a strict chronological experimental procedure. The overall experimental workflow included animal screening and health examination, estrus synchronization and mating, singleton pregnancy confirmation, pre-experiment adaptation, formal grouping and treatment, cold exposure intervention, sample collection, and subsequent laboratory analyses.

First, a total of 50 healthy 3–4-year-old Mongolian ewes were selected. All ewes received unified deworming treatment and comprehensive clinical examination to exclude individuals with underlying diseases and ensure consistent health status before the experiment. Subsequently, uniform estrus synchronization was performed for all qualified ewes: progesterone sponges were inserted on 12 August, and combined injection of prostaglandin and equine serum gonadotropin was administered 11 days later. Synchronized estrus was successfully induced on 25 August. During the subsequent unified estrous cycle, artificial mating was carried out using three healthy adult Mongolian rams from 10 to 15 September. On day 45 after mating (approximately 30 October), ultrasonic diagnosis was implemented to precisely screen ewes with singleton pregnancy, eliminating twin-pregnant and non-pregnant individuals.

All screened singleton-pregnant ewes were raised under natural grazing conditions until approximately day 80 of gestation (5 December). To ensure consistent baseline conditions, 24 pregnant ewes with similar body weight and identical gestational status were preliminarily selected for the formal trial. This study adopted a completely randomized design (CRD). The 24 experimental ewes were randomly assigned into three treatment groups, with eight biological replicates in each group: warm indoor control group (Group C), outdoor cold exposure group (Group L), and outdoor cold exposure + NCG supplementation group (Group LN).

Prior to the formal experiment, all indoor warm housing and outdoor experimental facilities were thoroughly disinfected. The entire experimental period contained a 10-day pre-feeding adaptation period and a subsequent formal treatment period. The formal experiment began at approximately day 90 of gestation (15 December) and ended on the unified sampling day (14 February of the following year), covering the critical mid-to-late gestation stage of Mongolian ewes. During the experiment, the average temperature in the warm housing was 3.60 °C, while the outdoor temperature averaged −12.20 °C. According to local meteorological records, the outdoor temperature fluctuated from −21.40 °C to −3.50 °C during the trial period, accompanied by natural wind exposure. Light conditions were automatically controlled to simulate the outdoor environment photoperiod. Ewes from cold-exposed groups (Group L and Group LN) were housed outdoors for the whole formal experimental period with no wind-proof shelter available.

During the whole experimental period, all ewes were fed a standardized basal diet formulated according to the nutritional criteria for 50 kg single-lamb pregnant ewes specified in Nutritional Requirements for Meat Sheep (NY/T816—2021), processed into full mixed pellets. The dietary composition and nutritional levels are listed in Table 1. Each ewe was provided with 1.5 kg of pelleted basal diet per day as the quantified feed offer, with free access to clean drinking water throughout the experiment. NCG intervention was performed by oral administration of 2.65 g commercial NCG product (95% purity), which contains 2.5 g pure active NCG. This dosage was selected according to our previous studies on pregnant sheep. Each daily dose was fully dissolved in 10 mL of distilled water and administered orally to ewes in the LN group at 07:00 every morning. Ewes in Group C and Group L received an equal volume of distilled water daily to eliminate solvent interference.

At the end of the formal experimental period, all experimental ewes were humanely slaughtered following standard animal welfare guidelines [21]. Liver tissues of ewes and their corresponding fetuses were rapidly collected, placed in sterile enzyme-free cryotubes, and immediately stored at −80 °C for subsequent laboratory analyses. Notably, all transcriptomic and metabolomic analyses in this study were performed simultaneously using the same batch of frozen liver samples collected uniformly at the endpoint of the experiment. Specifically, six individual liver samples from each group were selected and used for transcriptome sequencing, metabolomics profiling, and qRT-PCR validation.

Timeline summary: August—Animal selection, deworming and estrus synchronization; September—Artificial mating; October—Pregnancy diagnosis and singleton-ewe screening; 5 December—Pre-experiment adaptation started (gestation day 80); 15 December—Onset of cold exposure and daily NCG treatment (gestation day 90); 14 February—Slaughter and maternal–fetal liver tissue sampling.

### 2.2. RNA-Seq and Bioinformatics Analysis of Liver

Total RNA was extracted from the samples using TRIzol reagent (Invitrogen, Waltham, CA, USA) according to the manufacturer’s instructions and evaluated for concentration and purity [22]. Subsequently, qualified total RNA samples were used to synthesize cDNA using reverse transcriptase (Vazyme, Nanjing, China), and the samples were frozen at −20 °C for future use.

Following quality checks, sequencing was performed in Biomarker Technologies Corporation (Beijing, China). Each sample obtained approximately 6 G clean sequencing data, and the average genome mapping rate was above 90% (Tables S1 and S2). High-quality clean reads were filtered from the sequencing results and compared against databases to identify differential lncRNAs and miRNAs, along with their predicted target genes.

The sequencing results were analyzed to select differential genes, followed by GO and pathway enrichment analyses to preliminarily determine the biological functions of the differential lncRNAs and miRNAs. The interactions between lncRNAs and miRNAs were predicted, leading to the construction of a co-expression network for lncRNA-miRNA-mRNA. Key lncRNAs and miRNAs targeting the mTOR signaling pathway were identified, and several groups from the screening results were randomly selected for qRT-PCR validation, enabling relative quantitative analysis of gene expression.

#### 2.2.1. Differential lncRNA, mRNA, circRNA and miRNA Screening and Clustering Analysis

All liver tissue samples were individually sequenced without pooling. Whole-transcriptome libraries were constructed using the Ribo-Zero rRNA Removal Kit for rRNA depletion, and paired-end sequencing was performed on the Illumina NovaSeq platform with a read length of 150 bp. Clean reads were aligned to the latest Ovis aries reference genome (Oar_rambouillet_v1.0) using HISAT2 software (V 2.2.0), and transcript assembly was subsequently conducted using StringTie software (V 2.2.2). Transcript abundance of mRNAs, lncRNAs, circRNAs, and miRNAs was quantified using StringTie and RSEM software(V 1.3.3) [23], and the expression level of each transcript was normalized and calculated as fragments per kilobase of exon per million mapped fragments (FPKM). Differential expression analysis of coding and non-coding transcripts was performed using DESeq2 (V 1.42.x) [24]. In this study, transcripts with a false discovery rate (FDR) < 0.05 and |fold change| ≥ 1.5 were defined as significantly differentially expressed RNAs (DE RNAs), including up- and downregulated mRNAs, lncRNAs, circRNAs, and miRNAs. Based on the obtained differential profiles, Venn analysis was conducted to identify overlapping and specific DE RNAs across different comparison groups. The overall distribution of up- and ddownregulatedDE RNAs was visualized by colored pie charts. For expression pattern clustering, hierarchical clustering analysis was performed using Cluster 3.0 based on the normalized FPKM values of all DE RNAs, and the clustering heatmap was generated via TreeView v1.5, in which blue-white color indicates low expression and red color indicates high expression.

#### 2.2.2. GO Pathway and KEGG Enrichment Analysis

DAVID (Database for Annotation, Visualization and Integrated Discovery) was employed to predict the biological functions of lncRNAs via Gene Ontology (GO) functional annotation of their co-expressed target genes. GO terms were categorized into biological process (BP), cellular component (CC), and molecular function (MF). GO terms with *p* < 0.05 were screened, and Venn analysis was applied to identify shared GO terms. The top 10 enriched GO terms, ranked by fold enrichment and enrichment score, were exhibited. KEGG pathway analysis was further performed to characterize the biological pathways involving these co-expressed genes, and Venn analysis was used to obtain common pathways for up- and downregulated co-expressed genes. The GO, (http://www.geneontology.org/) and Kyoto Encyclopedia of Genes and Genomes (KEGG) [25] databases were utilized to interpret the biological roles of differentially expressed genes (DEGs). GO and KEGG terms with corrected *p* < 0.05 were regarded as statistically significant. Protein–protein interaction (PPI) networks of DEGs were constructed using STRING v10 [26], and the resulting network files were visualized in Cytoscape software (v3.7.1) [27] to exhibit biological interactions of core and hub genes. For lncRNA-mRNA regulatory network construction, protein-coding genes located within 100 kb upstream and downstream of each lncRNA were defined as potential cis-target genes. Three types of lncRNA-mRNA regulatory relationships, including antisense regulation, cis-regulation (adjacent to lncRNA loci), and trans-regulation (based on co-expression correlation), were predicted according to complementary pairing, neighboring position, and co-expression profiles [28].

#### 2.2.3. lncRNA-mRNA and circRNA-miRNA Co-Expression Network

The lncRNA-mRNA co-expression networks were built based on time dependent positive or negative correlations according to the normalized signal intensity of individual transcripts. The data were preprocessed by using the median gene expression intensity of all transcripts expressed from the same coding gene, without special treatment of the lncRNA expression value. Differentially expressed lncRNAs and mRNAs were then screened and removed from the dataset. Pearson’s correlation coefficient value was calculated for lncRNA–mRNA pairs, and strongly correlated pairs (0.8 or greater) were included (either positive or negative) in the co-expression network [29]. A *p*-value of less than 0.05 was considered statistically significant. A total of 241 lncRNAs and mRNAs containing 334 relationships were selected to generate a network map with cytoscape software (V. 3.2.1). Circle nodes represent lncRNAs and square nodes represent mRNAs. Red color and green color represent up and down regulation respectively. The darkness of the red and green shades represents fold changes in lncRNAs. The size of the circle represents *p*-value with larger sizes showing smaller *p*-values.

Similar to the lncRNA–mRNA network construction, the circRNA–miRNA co-expression network was constructed based on the correlation analysis between the differentially expressed circRNA and miRNAs. The expressions of differentially expressed circRNAs and miRNAs were analyzed using Pearson’s correlation coefficient. The absolute coefficient value of 0.8 between a circRNA and a miRNA was considered relevant for network construction. A *p*-value of less than 0.05 was considered statistically significant. A total of 24 circRNAs and 82 miRNAs containing 95 relationships were selected to generate a network map. A diamond node represents a circRNA and a circle node represents a miRNA. The colors yellow and blue represent up and down regulation respectively. The size of the diamonds represents fold change in circRNAs with larger sizes indicating higher fold change.

### 2.3. Metabolomics Analysis of Liver

#### 2.3.1. Metabolites Extraction

The LC/MS system for metabolomics analysis is composed of a Waters Acquity I-Class PLUS (Waters, Milford, CT, USA) ultra-high performance liquid tandem and a Waters Xevo G2-XS QTOF (Waters, Milford, CT, USA) high-resolution mass spectrometer. The column used was a Waters Acquity UPLC HSS T3 column (1.8 um 2.1 × 100 mm) (Waters, Milford, CT, USA). Positive ion mode: mobile phase A: 0.1% formic acid aqueous solution; mobile phase B: 0.1% formic acid acetonitrile. Negative ion mode: mobile phase A: 0.1% formic acid aqueous solution; mobile phase B: 0.1% formic acid acetonitrile. Injection volume 1 μL.

#### 2.3.2. LC-MS Analysis

The Waters Xevo G2-XS QTOF high-resolution mass spectrometer can collect primary and secondary mass spectrometry data in the MSe mode under the control of the acquisition software (MassLynx V4.2, Waters). In each data acquisition cycle, dual-channel data acquisition can be performed on both low collision energy and high collision energy at the same time. The low collision energy is 2 V, the high collision energy range is 10~40 V, and the scanning frequency is 0.2 s for a mass spectrum. The parameters of the ESI ion source are as follows: Capillary voltage: 2000 V (positive ion mode) or −1500 V (negative ion mode); cone voltage: 30 V; ion source temperature: 150 °C; desolvent gas temperature 500 °C; backflush gas flow rate: 50 L/ h; Desolventizing gas flow rate: 800 L/h.

#### 2.3.3. Data Preprocessing and Annotation

The raw data collected using MassLynx V4.2 is processed by Progenesis QI software (V 2.0) for peak extraction, peak alignment and other data processing operations, based on the Progenesis QI software online METLIN database and Biomark’s self-built library for identification, and at the same time, theoretical fragment identification and mass deviation. All are within 100 ppm.

#### 2.3.4. Data Analysis

After normalizing the original peak area information with the total peak area, the follow-up analysis was performed. Principal component analysis and Spearman correlation analysis were used to judge the repeatability of the samples within group and the quality control samples. The identified compounds were searched for classification and pathway information in KEGG, HMDB and lipidmaps databases. According to the grouping information, difference multiples were calculated and compare;, T test was used to calculate the difference in the significance pvalue of each compound. The R language package ropls was used to perform OPLS-DA modeling, and 200 times permutation tests were performed to verify the reliability of the model. The VIP value of the model was calculated using multiple cross-validation. The method of combining the difference multiple, the *p* value and the VIP value of the OPLS-DA model was adopted to screen the differential metabolites. The screening criteria were FC > 1, *p* value < 0.05 and VIP > 1. The differential metabolites of KEGG pathway enrichment significance were calculated using hypergeometric distribution test.

### 2.4. Quantitative Real-Time PCR (qRT-PCR) Assay

Candidate target genes were validated with highly reliable biotechniques such as qRT-PCR. Total RNA extraction and cDNA synthesis were performed as described previously [28]. Primers were designed and evaluated using the NCBI database. The primer efficiency was analyzed by standard curve. When the PCR amplification efficiency was between 0.9 and 1.1, it was used for qRT-PCR. Primer sequences of qRT-PCR are listed in Table 2. All qRT-PCR reactions were performed in the QuantStudioTM 5 system using SYBR Green master mix (cat. Q711, Vazyme, Nanjing, China), following the manufacturer’s instructions. The reaction system was 20 μL. Relative mRNA expression was calculated with the 2^−ΔΔCt^ method and normalized to *β-Actin* (ACTB) as an internal reference.

### 2.5. Statistical Analysis

All statistical analyses in this study were performed based on biological replicates from a single independent in vivo animal experiment. All quantitative data are presented as the mean ± standard error of the mean (SEM). All data were normally distributed, and variance was similar between the statistically compared groups. Statistical analyses were performed using GraphPad Prism 8.2 (GraphPad, San Diego, CA, USA) and SPSS software, version 24.0. (IBM, Armonk, NY, USA). A Student’s t test (two-tailed) or one-way analysis of variance (ANOVA) was performed followed by the Student–Newman–Keuls (SNK) method for multiple comparisons. Statistical significances were set at *p* values < 0.05.

## 3. Results

### 3.1. Identification of lncRNAs and mRNAs in the Liver of Cold-Stressed Mongolian Ewes and Fetuses Fed NCG

**Figure 1:**
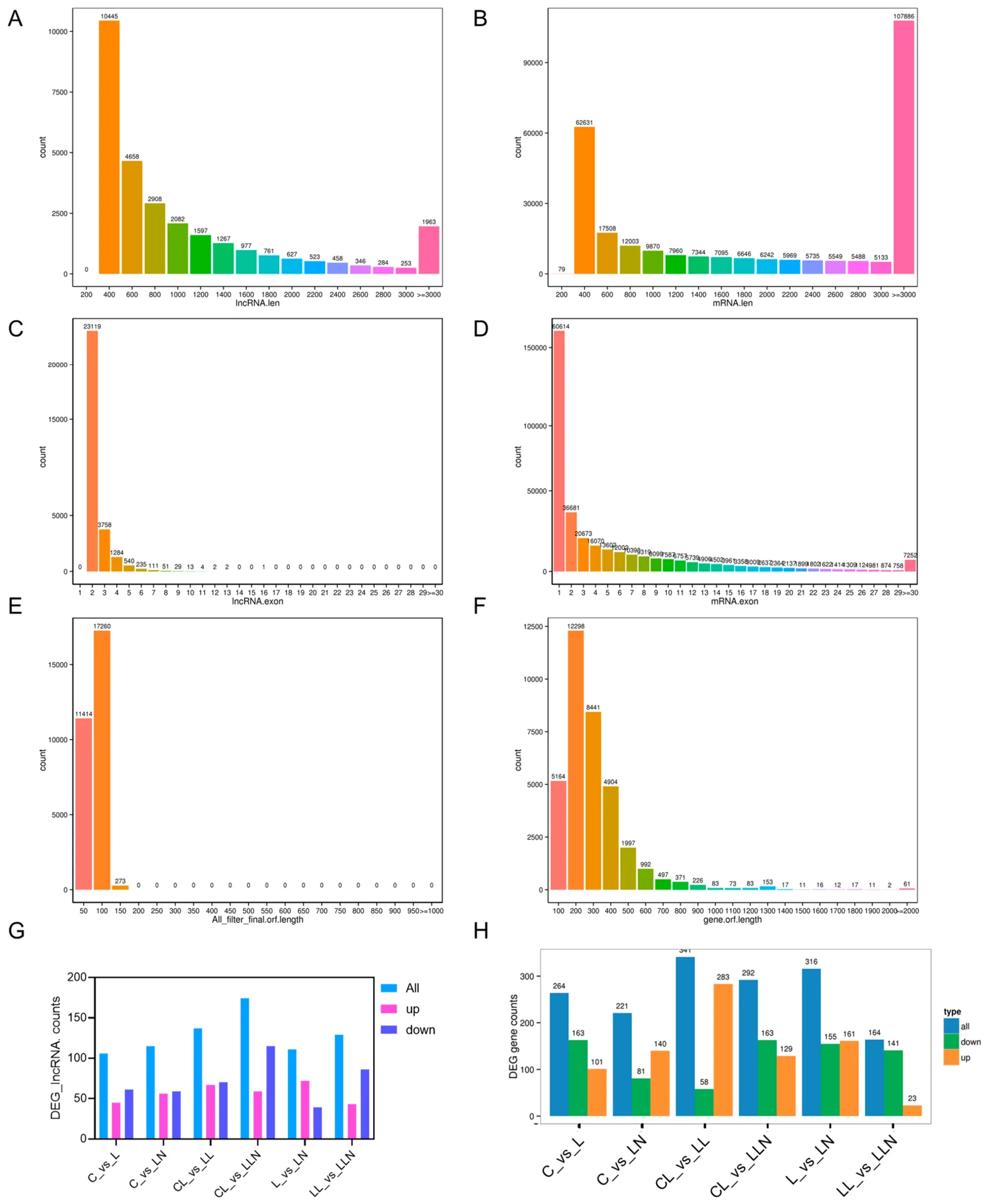
Identification of lncRNAs and mRNAs in the liver of cold-stressed Mongolian ewes and fetuses fed with NCG. (**A**,**B**) Length distribution of lncRNAs and mRNAs. (**C**,**D**) Exon number distribution of mRNAs and lncRNAs. (**E**,**F**) ORF length of lncRNAs and mRNAs. (**G**,**H**) Histogram of the number of DE mRNAs and lncRNAs in the liver of cold-stressed Mongolian ewes and fetuses fed NCG.

In total, we sequenced the liver tissue of cold-stressed Mongolian ewes and fetuses feeding with N-Carbamylglutamate (NCG). LncRNA lengths were mainly distributed in the range of 400–3000 and >3000 bp, consistent with those of mRNA transcripts (Figure 1A,B). LncRNAs mainly contained 2–13 exons, which was significantly fewer than the exons of mRNA transcripts (Figure 1C,D). The open reading frame (ORF) length of lncRNA transcripts was shorter than that of the mRNA transcripts (Figure 1E,F). Furthermore, the number of differentially expressed lncRNAs (DE lncRNA) identified in C vs. L group, C vs. LN group, L vs. LN group, CL vs. LL group, CL vs. LLN group and LL vs. LLN group were 106 (45 upregulated and 61 downregulated), 115 lncRNAs (56 upregulated and 59 downregulated), 111 (72 upregulated and 39 downregulated), 137 lncRNAs (67 upregulated and 70 downregulated), 174 lncRNAs (59 upregulated and 115 downregulated) and 129 lncRNAs (43 upregulated and 89 downregulated), respectively. And a total of 264 DE mRNAs (101 upregulated and 163 downregulated), 221 DE mRNAs (140 upregulated and 81 downregulated), 316 DE mRNAs (161 upregulated and 155 downregulated), 341 DE mRNAs (283 upregulated and 58 downregulated), 292 DE mRNAs (129 upregulated and 163 downregulated), and 164 DE mRNAs (23 upregulated and 141 downregulated) were identified in C vs. L group, C vs. LN group, L vs. LN group, CL vs. LL group, CL vs. LLN group, and LL vs. LLN group, respectively (Figure 1H).

### 3.2. Expression Analysis of lncRNAs and mRNAs in the Liver of Cold-Stressed Mongolian Ewes Fed NCG

**Figure 2:**
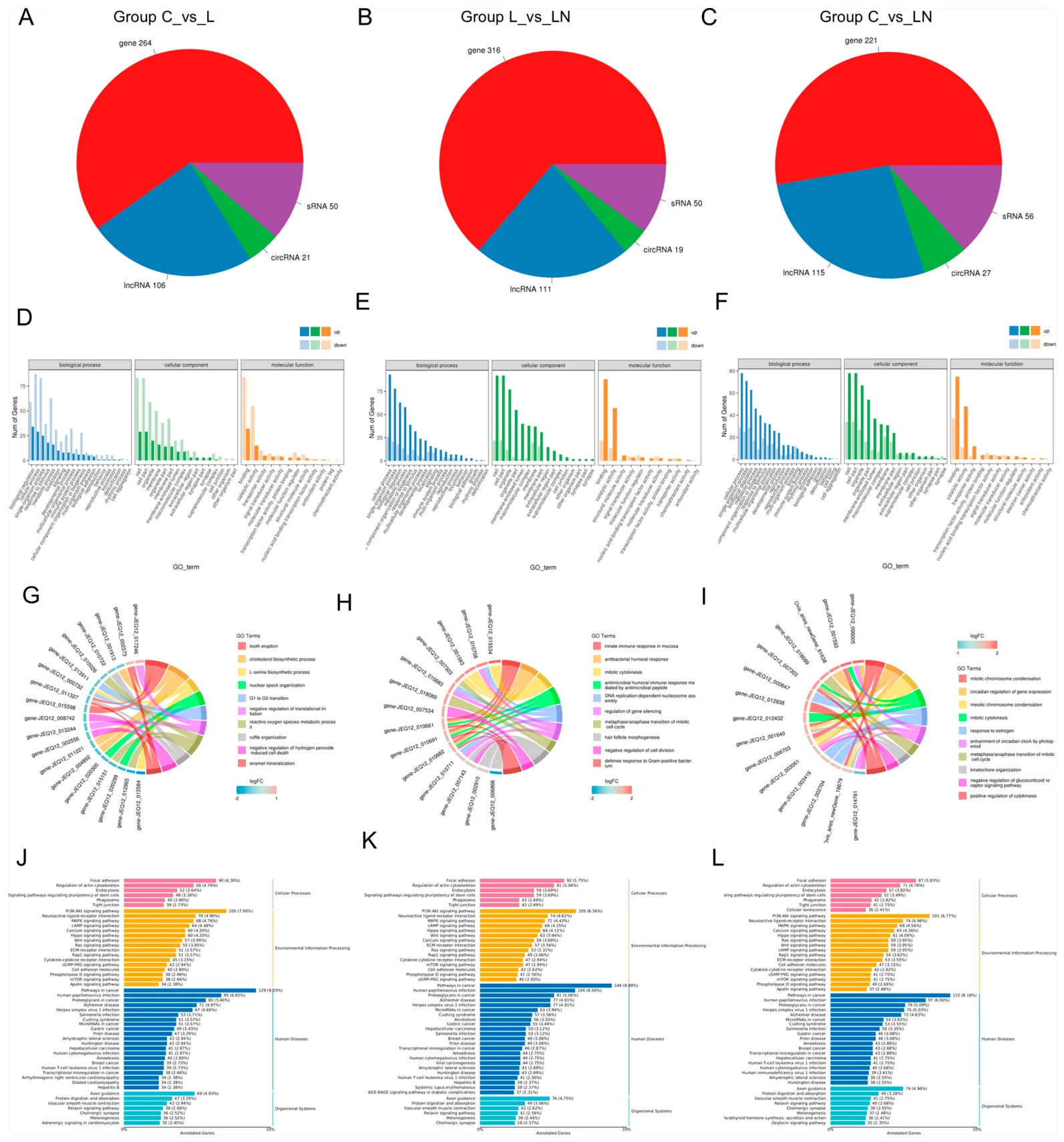
A distinct liver expression profile was identified in cold-stressed Mongolian ewes following the supplementation of N-carbamylglutamate (NCG). (**A**–**C**) Bin plot of the number of DE gene, lncRNAs, circRNA and sRNA between the different groups. (**D**–**F**) Histogram of GO function annotation (BP, CC and MF) of DE genes. (**G**–**I**) Circos plot of GO annotation with TOP 10 DE genes. (**J**–**L**) The diagrams of two significant signaling pathways by KEGG pathway enrichment.

A total of 264 differentially expressed genes, 106 differentially expressed lncRNAs, 21 differentially expressed circRNAs, and 50 sRNAs were identified between the control group and the cold stress group (C vs. L) (Figure 2A). GO functional enrichment analysis of the differentially expressed genes revealed that, at the BP level, they were primarily enriched in biological regulation, cellular processes, and single-organism processes. At the CC level, they were mainly enriched in cell, cell part, and organelle, while at the MF level, they were primarily enriched in binding, catalytic activity, and transporter activity (Figure 2D). The top 10 enriched GO terms were selected to create an enrichment chord diagram, with significantly enriched terms including cholesterol biosynthetic process, L-serine biosynthetic process, G1 to G0 transition, reactive oxygen species metabolic process, and negative regulation of hydrogen peroxide-induced cell death (Figure 2G). Subsequently, KEGG functional enrichment analysis indicated that the differentially expressed genes were mainly enriched in pathways such as focal adhesion, PI3K/AKT, MAPK, cAMP, Hippo, Wnt, Ras, and mTOR signaling pathways, closely related to pathways in human cancers (Figure 2J).

A total of 316 differentially expressed genes, 111 differentially expressed lncRNAs, 19 differentially expressed circRNAs, and 50 sRNAs were identified between the cold stress group and the NCG supplementation group (L vs. LN) (Figure 2B). GO functional enrichment analysis revealed that, at the BP level, the differentially expressed genes were primarily enriched in biological regulation, cellular processes, and single-organism processes. At the CC level, they were mainly enriched in cell, cell part, and organelle, while at the MF level, they were primarily enriched in binding, catalytic activity, and structural molecule activity (Figure 2F). The top 10 enriched GO terms were selected for an enrichment chord diagram, with significantly enriched terms including innate immune response in mucosa, antibacterial humoral response, mitotic cytokinesis, regulation of gene silencing, and defense responses to Gram-positive bacteria (Figure 2H). KEGG functional enrichment analysis revealed that the differentially expressed genes were primarily enriched in focal adhesion, PI3K/AKT, MAPK, cAMP, Hippo, Wnt, Ras, and mTOR signaling pathways, closely related to pathways in human cancers (Figure 2K).

Furthermore, this study analyzed the effects of NCG supplementation on liver tissues of ewes under cold exposure during late pregnancy. In the comparison between the control group and the NCG supplemented group (C vs. LN), a total of 221 differentially expressed genes, 115 differentially expressed lncRNAs, 27 differentially expressed circRNAs, and 56 sRNAs were identified (Figure 2C). GO functional enrichment analysis showed that, at the BP level, the differentially expressed genes were primarily enriched in biological regulation, cellular processes, and single-organism processes. At the CC level, they were mainly enriched in cell, cell part, and organelle, while at the MF level, they were primarily enriched in binding, catalytic activity, and transporter activity (Figure 2E). The top 10 enriched GO terms were selected for an enrichment chord diagram, with significantly enriched terms including circadian regulation of gene expression, mitotic cytokinesis, response to estrogen, and positive regulation of cytokinesis (Figure 2I). KEGG functional enrichment analysis indicated that the differentially expressed genes were primarily enriched in focal adhesion, PI3K/AKT, MAPK, cAMP, Hippo, Wnt, Ras, and mTOR signaling pathways, closely related to pathways in cancer in human diseases (Figure 2L).

### 3.3. Expression Analysis of lncRNAs and mRNAs in the Liver of Cold-Stressed Mongolian Fetuses Feeding with NCG

**Figure 3:**
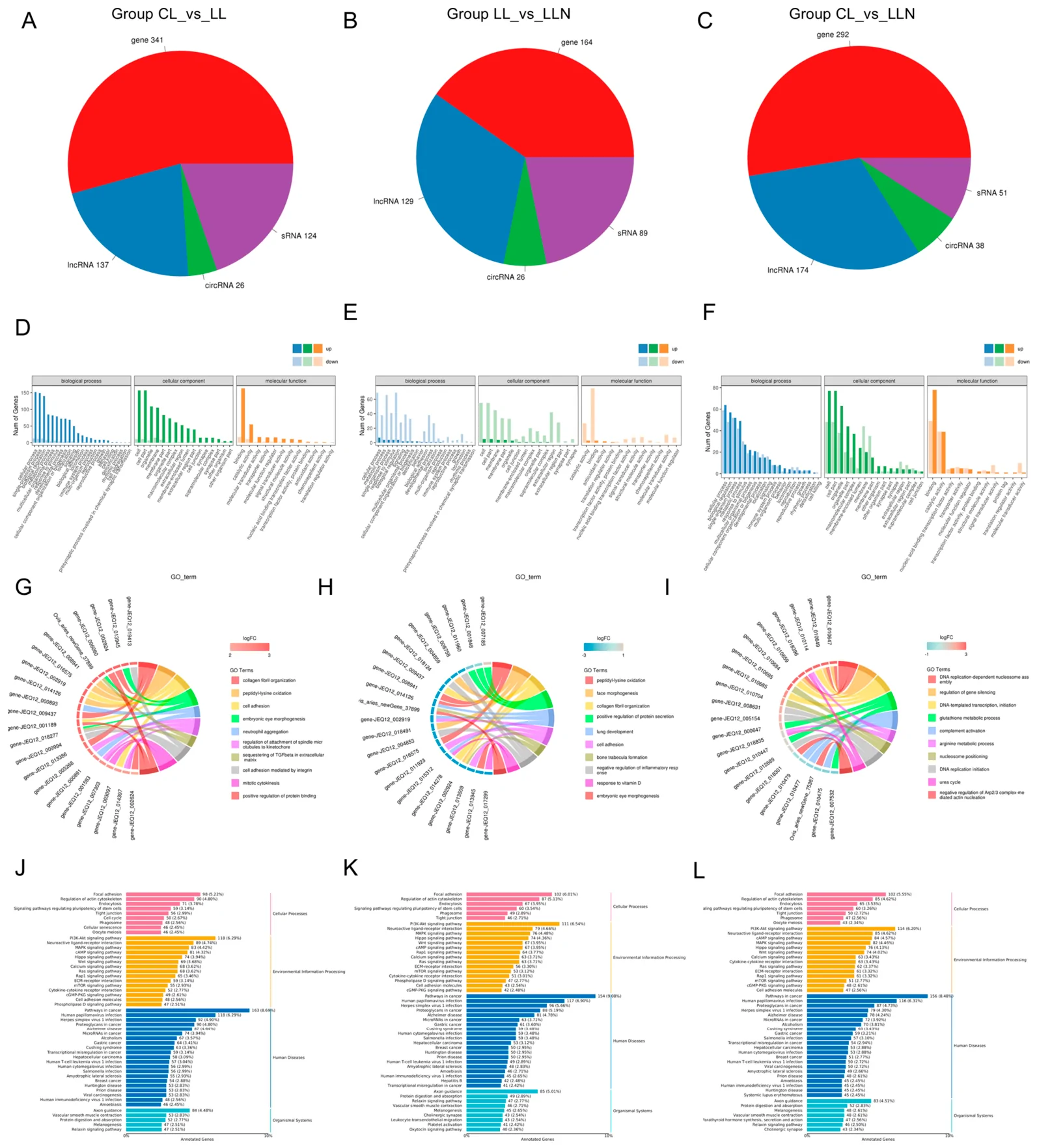
A distinct liver expression profile was identified in cold-stressed Mongolian fetuses following the supplementation of N-carbamylglutamate (NCG). (**A**–**C**) Bin plot of the number of DE gene, lncRNAs, circRNA and sRNA between the different groups. (**D**–**F**) Histogram of GO function annotation (BP, CC and MF) of DE genes. (**G**–**I**) Circos plot of GO annotation with TOP 10 DE genes. (**J**–**L**) The diagrams of two significant signaling pathways by KEGG pathway enrichment.

A total of 341 differentially expressed genes, 137 differentially expressed lncRNAs, 25 differentially expressed circRNAs, and 124 sRNAs were identified between the control group and the cold stress group (CL vs. LL) (Figure 3A). GO functional enrichment analysis of the differentially expressed genes revealed that, at the BP level, they were primarily enriched in cellular processes, single-organism processes, and biological regulation. At the CC level, they were mainly enriched in cell, cell part, and organelle, while at the MF level, they were predominantly enriched in binding and catalytic activity (Figure 3D). The top 10 enriched GO terms were selected to create an enrichment chord diagram, with significantly enriched terms including collagen fibril organization and peptidyl-lysine oxidation (Figure 3G). Subsequent KEGG functional enrichment analysis indicated that the differentially expressed genes were primarily enriched in focal adhesion, PI3K/AKT, MAPK, cAMP, Hippo, Wnt, Ras, and mTOR signaling pathways, closely related to pathways in cancer in human diseases (Figure 3J).

A total of 164 differentially expressed genes, 129 differentially expressed lncRNAs, 26 differentially expressed circRNAs, and 39 sRNAs were identified between the cold stress group and the NCG supplementation group (LL vs. LLN) (Figure 3B). GO functional enrichment analysis revealed that, at the BP level, the differentially expressed genes were primarily enriched in cellular processes, metabolic processes, single-organism processes, and response to stimulus. At the CC level, they were mainly enriched in cell, cell part, membrane, and membrane part, while at the MF level, they were predominantly enriched in binding and catalytic activity (Figure 3E). The top 10 enriched GO terms were selected for an enrichment chord diagram, with significantly enriched terms including peptidyl-lysine oxidation, face morphogenesis, and collagen fibril organization (Figure 3H). KEGG functional enrichment analysis indicated that the differentially expressed genes were primarily enriched in focal adhesion, PI3K/AKT, MAPK, cAMP, Hippo, Wnt, Ras, and mTOR signaling pathways, closely related to pathways in cancer in humans. (Figure 3K).

In the comparison between the control group and the NCG supplemented group (CL vs. LLN), a total of 292 differentially expressed genes, 174 differentially expressed lncRNAs, 38 differentially expressed circRNAs, and 51 sRNAs were identified (Figure 3C). GO functional enrichment analysis showed that, at the BP level, the differentially expressed genes were primarily enriched in cellular processes, biological regulation, single-organism processes, and metabolic processes. At the CC level, they were mainly enriched in cell, cell part, and organelle, while at the MF level, they were predominantly enriched in binding and catalytic activity (Figure 3F). The top 10 enriched GO terms were selected for an enrichment chord diagram, with significantly enriched terms including DNA replication-dependent nucleosome assembly, regulation of gene silencing, and DNA-templated transcription initiation (Figure 3I). KEGG functional enrichment analysis indicated that the differentially expressed genes were primarily enriched in focal adhesion, PI3K/AKT, MAPK, cAMP, Hippo, Wnt, Ras, and mTOR signaling pathways, closely related to pathways in human cancers (Figure 3L).

### 3.4. Integrated Analysis of Differential RNA Targets in Cold-Stressed Mongolian Ewes and Fetuses Following NCG Supplementation

**Figure 4:**
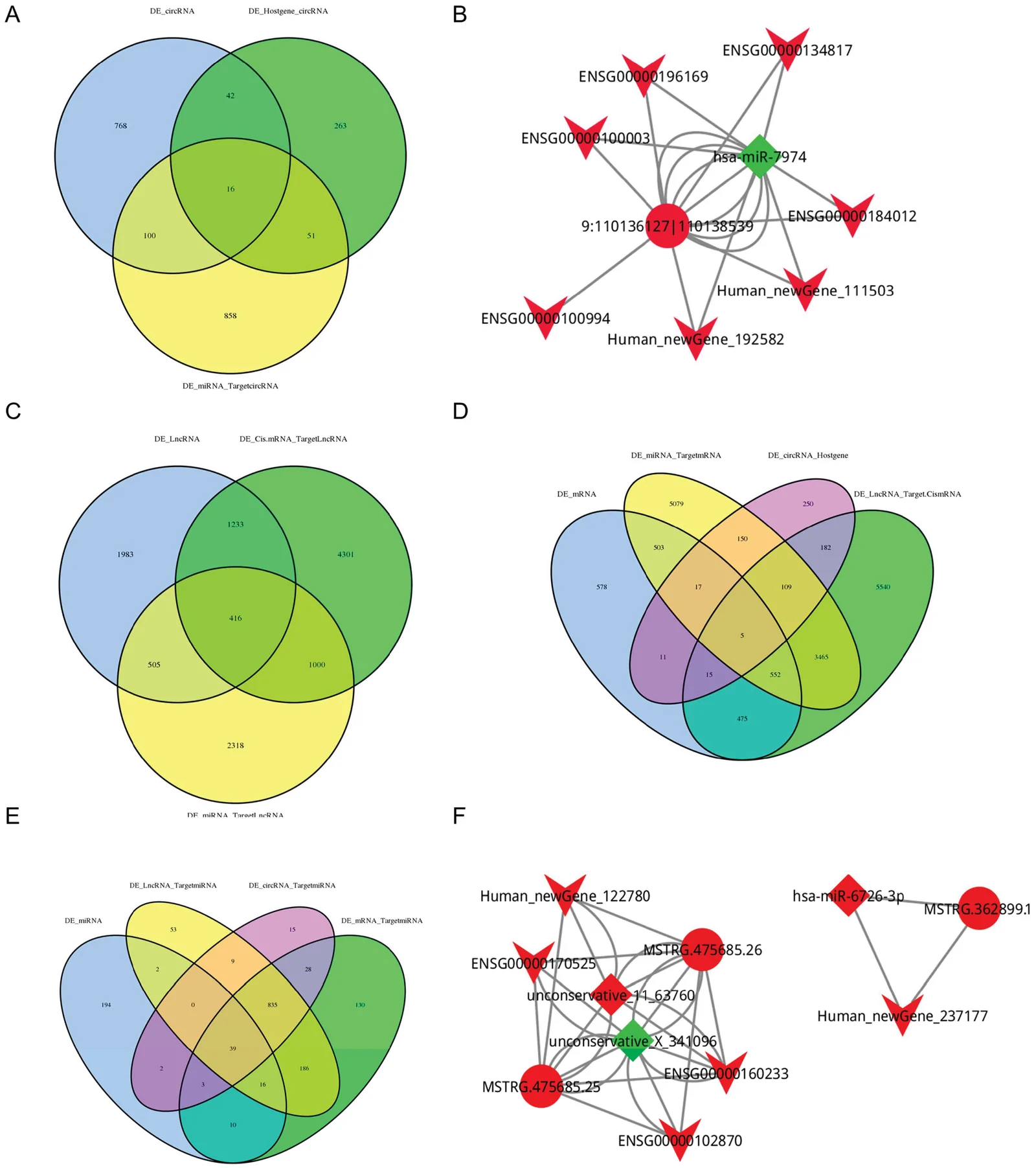
Integrated analysis of differential RNA targets in cold-stressed Mongolian ewes and fetuses following NCG supplementation. (**A**) Venn diagram of differential circRNA–miRNA–mRNA; (**B**) Example of differential circRNA–miRNA–mRNA relationships based on ceRNA network; (**C**) Venn diagram of cis-regulatory relationships in the differential lncRNA–miRNA–mRNA network; (**D**) Venn diagram analyzing cis-regulatory relationships in differential circRNA–miRNA–mRNA relationships; (**E**) Venn diagram analyzing differential circRNA–lncRNA–miRNA–mRNA relationships based on the ceRNA network; (**F**) Example of differential lncRNA–miRNA–mRNA relationships. Note: In the diagrams, lncRNA, miRNA, and mRNA are represented by circles, diamonds, and arrows, respectively, with upregulation and downregulation indicated in red and green.

A systematic screening of their upstream and downstream targeting interactions was conducted, particularly incorporating the host gene information corresponding to circRNA to ensure the integrity and biological significance of the regulatory network. Venn analysis results (Figure 4A,C) revealed a substantial number of co-targeted RNA molecules within the potential targeting relationship pools of differential circRNA–miRNA–mRNA (Figure 4A) and differential lncRNA–miRNA–mRNA (Figure 4C). Based on the intersections identified in the Venn diagram, this study constructed ceRNA regulatory networks for circRNA–miRNA–mRNA and lncRNA–miRNA–mRNA, visualized using Cytoscape software to systematically analyze the interaction patterns between non-coding RNAs and mRNAs (Figure 4B). For the lncRNA-related regulatory network, we not only examined the ceRNA mechanism but also emphasized the selection of cis-regulatory relationships (Figure 4D–F). The Venn diagram (Figure 4D) clarifies the intersection of key genes involved in both ceRNA and cis-regulation, further refining the regulatory patterns of lncRNA. The visualized network (Figure 4F) illustrates the targeted connections among lncRNA (represented by circular nodes), miRNA (represented by diamond nodes), and mRNA (indicated by arrow nodes). This network not only reflects the regulation of mRNA expression by lncRNA through ceRNA mechanisms but also highlights the biological processes by which lncRNA regulates host genes or related genes in adjacent regions. The differently colored nodes and connecting lines distinctly present the expression synergy and regulatory directionality of upstream and downstream molecules.

### 3.5. Hepatic Metabolomic Profiling of Cold-Stressed Mongolian Ewes with NCG Supplementation

**Figure 5:**
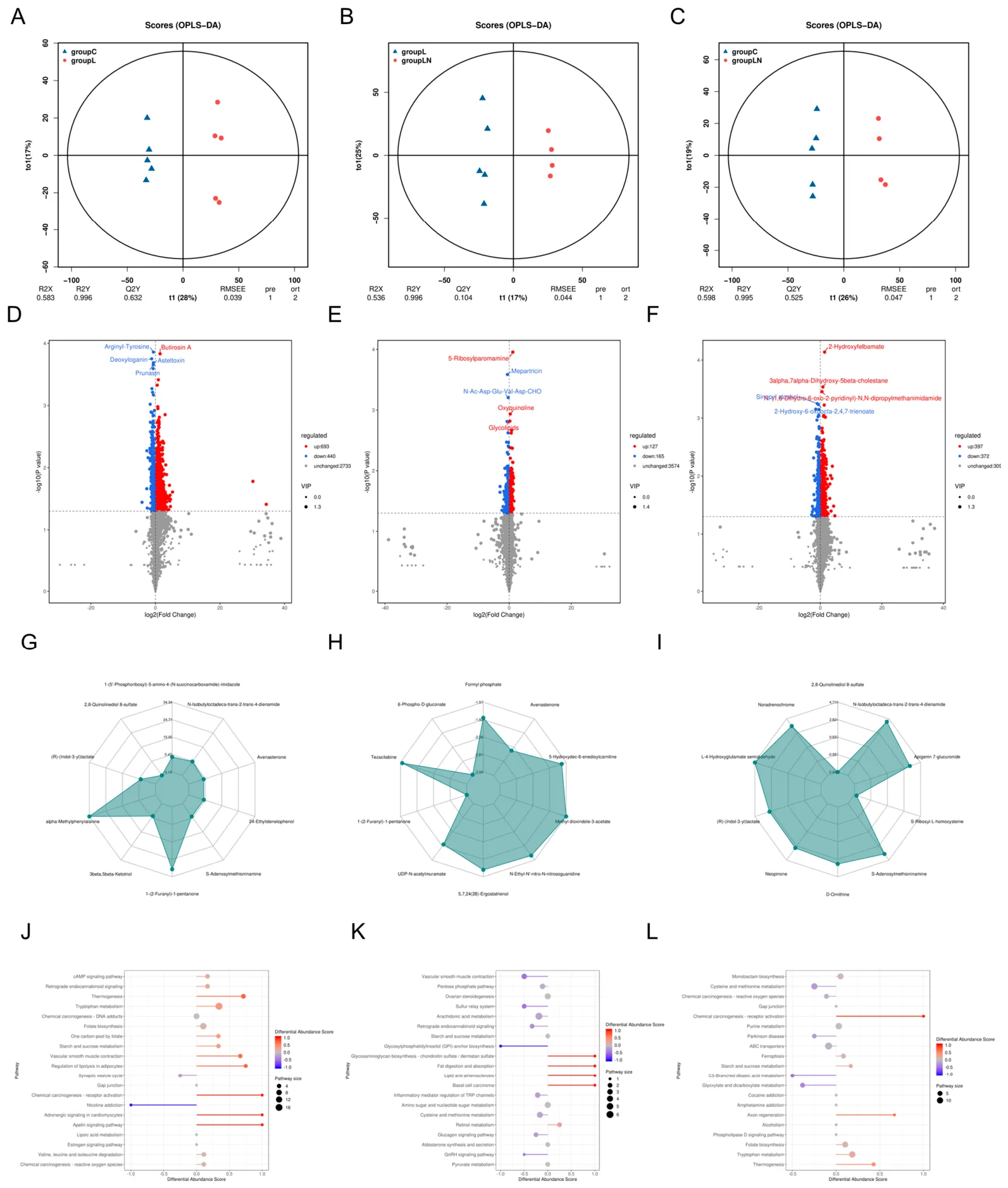
Metabolomic profiling of the liver in cold-stressed Mongolian ewes fed NCG. (**A**–**C**) OPLS-DA score plots of pairwise comparisons: (**A**) group C vs. group L, (**B**) group L vs. group LN, (**C**) group C vs. group LN. Model parameters (R2X, R2Y, Q2Y, RMSEE) are listed below each plot, demonstrating good model fitness and predictive power. (**D**–**F**) Volcano plots of differential metabolites: (**D**) C vs. L, (**E**) L vs. LN, (**F**) C vs. LN. Red dots indicate upregulated metabolites, blue dots indicate downregulated metabolites (VIP > 1, *p* < 0.05), and gray dots represent non-significant metabolites. Key differential metabolites are annotated. (**G**–**I**) Radar charts displaying the relative abundance of top differential metabolites in each comparison: (**G**) C vs. L, (**H**) L vs. LN, (**I**) C vs. LN. (**J**–**L**) KEGG pathway enrichment analysis of differential metabolites: (**J**) C vs. L, (**K**) L vs. LN, (**L**) C vs. LN. The color gradient represents the differential abundance score, and the dot size represents the number of enriched metabolites.

After quality control of the OPLS-DA model, differential metabolites were identified in liver samples from Mongolian sheep. OPLS-DA analysis demonstrated distinct classification of metabolites between C, L, and LN groups (Figure 5A). Differentially expressed metabolites were screened in the liver tissues of Mongolian sheep, meeting criteria of *p*-value < 0.05, VIP >= 1, and fold change < 0.67 or >1.5, as illustrated in volcano plots (Figure 5B). A total of 693 upregulated and 440 downregulated differential metabolites were found to be significantly expressed in C vs. L groups. Among them, the abundance of Arginyl-tyrosine, Asteltoxin, Deoxyloganin, Prunasin was significantly lower, but Butirosin A was higher in the L group than the C group. For L vs. LN groups, 127 upregulated and 165 downregulated differential metabolites were screened, including 5-Ribosylparomamine, Mepartricin, Oxyquinoline and Glycolipids. Furthermore, 397 upregulated and 372 downregulated differential metabolites were identified in the LN groups relative to the control groups, including 2-Hydroxyfelbamate and Sinapyl alcohol. To characterize the metabolic differences between groups, we performed untargeted metabolomic analysis and constructed OPLS-DA models for pairwise comparisons. The OPLS-DA score plots (Figure 5A–C) showed clear separation between each pair of groups, with high R2Y and Q2Y values (all > 0.98), confirming the reliability of the models and distinct metabolic profiles among groups. Volcano plot analysis (Figure 5D–F) identified a set of significantly differential metabolites (VIP > 1, *p* < 0.05) in each comparison. In the C vs. L comparison (Figure 5D), key differential metabolites included Butirosin A, Arginyl-Tyrosine, Deoxyloganin and Asteltoxin. In the L vs. LN comparison (Figure 5E), notable changes were observed in 5-Ribosylparomamine and Mepartricin. In the C vs. LN comparison (Figure 5F), 2-Hydroxyfelbamate, and 3alpha, 7alpha-Dihydroxy-5beta-cholestane were among the top differential metabolites. Radar charts (Figure 5G–I) further visualized the relative abundance of these key differential metabolites, highlighting the most prominent changes in each comparison group. KEGG pathway enrichment analysis (Figure 5J–L) revealed that differential metabolites were primarily enriched in pathways related to lipid metabolism, amino acid metabolism, and energy metabolism. These pathways are closely associated with cellular physiological processes, suggesting that the observed metabolic changes may play critical roles in the phenotypic differences between groups.

### 3.6. Hepatic Metabolomic Profiling of Fetal Liver from Cold-Stressed Mongolian Ewes with NCG Supplementation

**Figure 6:**
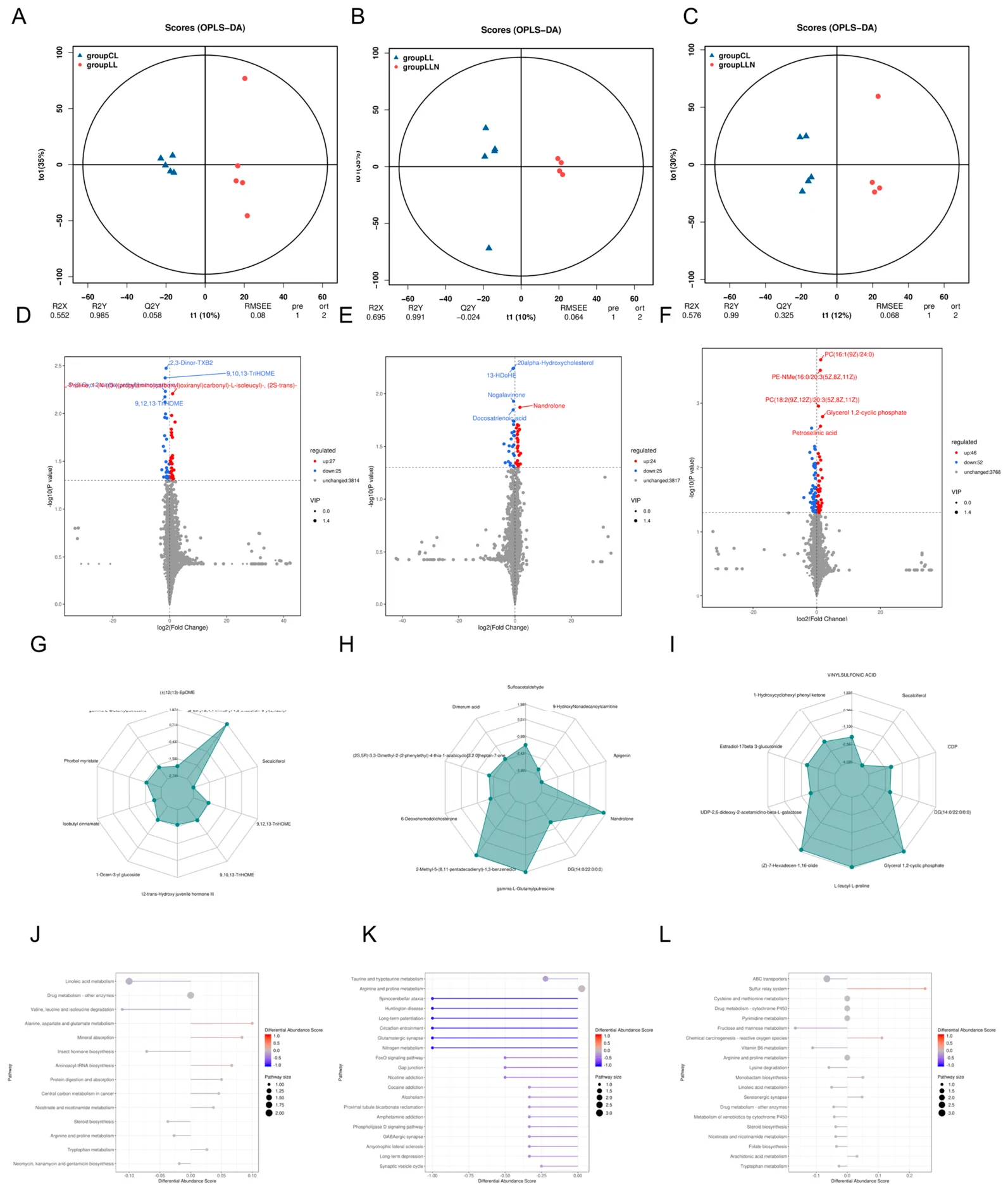
Metabolomic profiling reveals distinct metabolic signatures between CL, LL and LLN group. (**A**–**C**) OPLS-DA score plots of pairwise comparisons: (**A**) group CL vs. group LL, (**B**) group LL vs. group LLN, (**C**) group CL vs. group CLN. The model parameters (R2X, R2Y, Q2Y, RMSEE) are listed below each plot, indicating good model fitness and predictive ability. (**D**–**F**) Volcano plots of differential metabolites: (**D**) CL vs. LL, (**E**) LL vs. LLN, (**F**) CL vs. CLN. Red dots represent upregulated metabolites, blue dots represent downregulated metabolites (VIP > 1, *p* < 0.05), and gray dots represent non-significant metabolites. Key differential metabolites are labeled. (**G**–**I**) Radar charts showing the relative abundance of top differential metabolites in each comparison: (**G**) CL vs. LL, (**H**) LL vs. LLN, (**I**) CL vs. CLN. (**J**–**L**) KEGG pathway enrichment analysis of differential metabolites: (**J**) CL vs. LL, (**K**) LL vs. LLN, (**L**) CL vs. CLN. The color gradient represents the differential abundance score, and the dot size represents the number of enriched metabolites.

We performed untargeted metabolomic analysis and constructed OPLS-DA models for pairwise comparisons. The OPLS-DA score plots (Figure 6A–C) showed clear separation between each pair of groups, with high R2Y and Q2Y values (all > 0.98), confirming the reliability of the models and distinct metabolic profiles among groups. Volcano plot analysis (Figure 6D–F) identified a set of significantly differential metabolites (VIP > 1, *p* < 0.05) in each comparison. In the CL vs. LL comparison (Figure 6D), key differential metabolites included 2,3-Dinor-TXB2 and 12,13-Dihydroxy-9-octadecenoic acid. In the LL vs. LLN comparison (Figure 6E), notable changes were observed in 13-HODE and Docosahexaenoic acid. In the CL vs. CLN comparison (Figure 6F), PE (18:2(9Z,12Z)/18:2(9Z,12Z)) and Peroxynitric acid were among the top differential metabolites. Radar charts (Figure 6G–I) further visualized the relative abundance of these key differential metabolites, highlighting the most prominent changes in each comparison group. KEGG pathway enrichment analysis (Figure 6J–L) revealed that differential metabolites were primarily enriched in pathways related to lipid metabolism, amino acid metabolism, and energy metabolism. These pathways are closely associated with cellular physiological processes, suggesting that the observed metabolic changes may play critical roles in the phenotypic differences between groups.

### 3.7. Integrated Analysis of Liver Metabolomic Profiling in Cold-Stressed Mongolian Ewes and Fetuses Following NCG Supplementation

**Figure 7:**
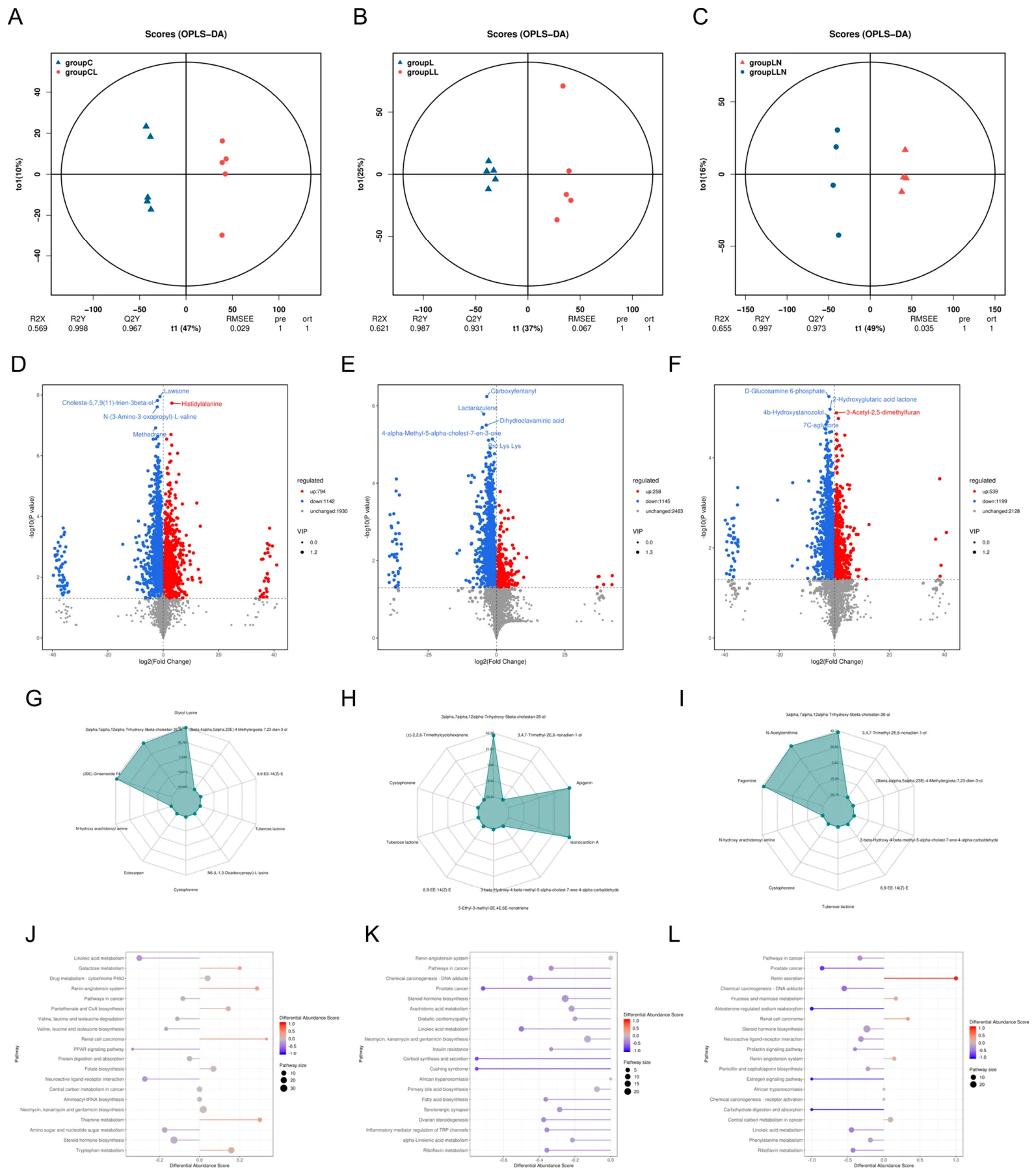
Integrated analysis of liver metabolomic profiling in cold-stressed Mongolian ewes and fetuses following NCG supplementation. (**A**–**C**) OPLS-DA score plots of pairwise comparisons: (**A**) group C vs. group CL, (**B**) group LL vs. group LLN, (**C**) group CL vs. group LLN. Model parameters (R2X, R2Y, Q2Y, RMSEE) are listed below each plot, demonstrating good model fitness and predictive power. (**D**–**F**) Volcano plots of differential metabolites: (**D**) C vs. CL, (**E**) LL vs. LLN, (**F**) CL vs. LLN. Red dots indicate upregulated metabolites, blue dots indicate downregulated metabolites (VIP > 1, *p* < 0.05), and gray dots represent non-significant metabolites. Key differential metabolites are annotated. (**G**–**I**) Radar charts displaying the relative abundance of top differential metabolites in each comparison: (**G**) C vs. CL, (**H**) LL vs. LLN, (**I**) CL vs. LLN. (**J**–**L**) KEGG pathway enrichment analysis of differential metabolites: (**J**) C vs. CL, (**K**) LL vs. LLN, (**L**) CL vs. LLN. The color gradient represents the differential abundance score, and the dot size represents the number of enriched metabolites.

This study employed non-targeted metabolomics to analyze liver samples from various groups and constructed an orthogonal partial least squares discriminant analysis (OPLS-DA) model. The integrated OPLS-DA score plots (Figure 7A–C) demonstrated clear separation among samples in the model, with model fit parameters (R^2^Y, Q^2^Y > 0.96) indicating robust discriminatory capability and predictive performance. This confirms that cold exposure and NCG intervention significantly altered the liver metabolic profiles. Differential metabolites were screened based on variable importance projection (VIP > 1) and t-tests (*p* < 0.05), with a volcano plot (Figure 7D–F) illustrating the distribution of these differential metabolites among groups, which will be explained below.

For the C vs. CL group (Figure 7D): cold exposure induced notable metabolic disturbances, with core differential metabolites including Crocin, N-(3-Amino-3-carboxypropyl)-S-valine, and Histidylalanine, suggesting alterations in amino acid metabolism, energy metabolism, and oxidative stress pathways. In the LL vs. LLN group (Figure 7E): NCG intervention significantly reversed the metabolic changes induced by cold exposure, with differential metabolites represented by 4-alpha-Methyl-5-alpha-cholest-7-en-3-one, Lactobionic acid, and Dihydroxydecanoic acid, indicating that NCG alleviates cold stress injury by regulating lipid metabolism and energy homeostasis pathways. In the CL vs. LLN group (Figure 7F): NCG intervention significantly regulated the metabolism of ewes under cold stress, with differential metabolites including D-Glucosamine 6-phosphate, 4b-Hydroxycholestan-3-one, and 3-Acetyl-2,5-dimethylfuran, suggesting that NCG improves metabolic imbalance under cold stress by modulating key pathways related to carbohydrate metabolism, lipid metabolism, and amino acid metabolism. Radar plots (Figure 7G–I) further quantified the relative abundance changes in core differential metabolites, clearly depicting the upregulation/downregulation trends of metabolites under cold exposure and NCG intervention. KEGG pathway enrichment analysis (Figure 7J–L) revealed that differential metabolites were primarily enriched in pathways related to amino acid metabolism, lipid metabolism, carbohydrate metabolism, and oxidative stress.

### 3.8. A Conjoint Analysis of Liver Transcriptome and Metabolome in Cold-Stressed Mongolian Ewes Following NCG Supplementation

**Figure 8:**
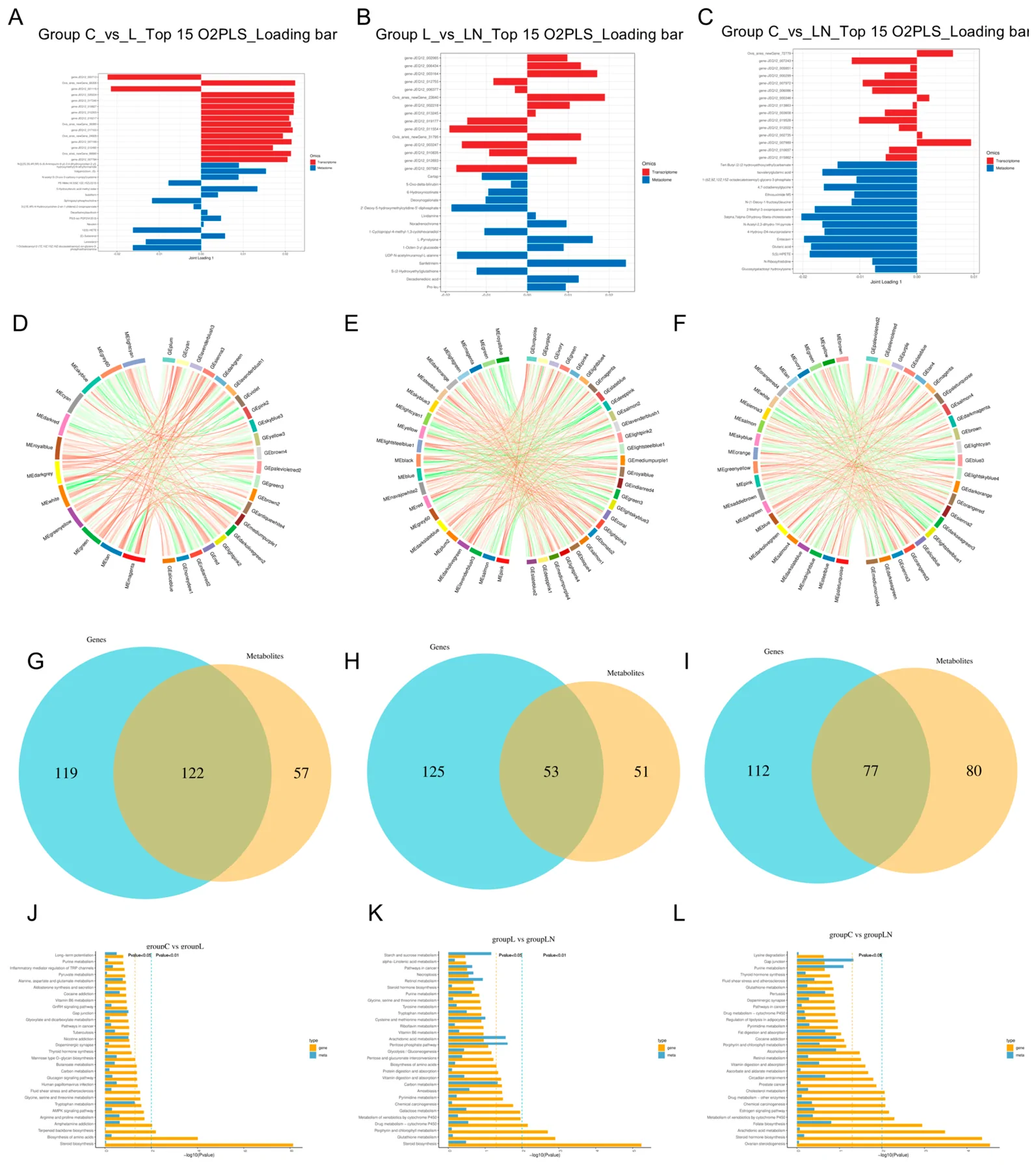
A Conjoint analysis of liver transcriptome and metabolome in cold-stressed Mongolian ewes following NCG supplementation. (**A**–**C**) Bar plots of the top 15 genes/metabolites with highest O2PLS loading values for pairwise comparisons: (**A**) group C vs. group L, (**B**) group L vs. group LN, (**C**) group C vs. group LN. Red and blue bars represent positive and negative correlations, respectively. (**D**–**F**) Chord diagrams illustrating the correlation network between key genes and metabolites for (**D**) C vs. L, (**E**) L vs. LN, (**F**) C vs. LN. Each color represents a functional category, and connecting lines indicate significant correlations between genes and metabolites. (**G**–**I**) Venn diagrams showing the overlap between significantly differentially expressed genes and differential metabolites for (**G**) C vs. L, (**H**) L vs. LN, (**I**) C vs. LN. (**J**–**L**) Correlation heatmaps of the top differential genes and metabolites for (**J**) C vs. L, (**K**) L vs. LN, (**L**) C vs. LN. The color gradient represents the correlation coefficient (red: positive, blue: negative).

Transcriptomic and metabolomic datasets were integrated for conjoint analysis. OPLS-DA loading plots (Figure 8A–C) showed clear separation among the C, L, LN and CL, LL and LLN groups, indicating distinct hepatic molecular profiles under cold stress and after NCG supplementation. Correlation network analysis (Figure 8D–F) revealed extensive positive and negative correlations between differentially expressed genes and differential metabolites in each pairwise comparison, suggesting that cold stress reshaped hepatic metabolic homeostasis through transcriptional regulation, and NCG partially remodeled these gene–metabolite association patterns. Venn diagrams (Figure 8G–I) further characterized shared and unique differential genes and metabolites: 122 overlapping gene–metabolite pairs were identified in C vs. CL, 53 in L vs. LL, and 77 in LN vs. LLN, demonstrating that cold stress induced broad transcriptomic and metabolomic perturbations while NCG reversed a portion of these alterations. Integrated pathway enrichment (Figure 8J–L) showed that pathways related to amino acid metabolism, energy metabolism, lipid metabolism, and oxidative stress were significantly enriched across comparisons. Cold stress disrupted hepatic amino acid and energy pathways, and NCG supplementation effectively restored part of these perturbed pathways. Collectively, the conjoint analysis indicates that NCG exerts hepatic protective effects against cold stress by modulating the crosstalk between gene transcription and metabolite homeostasis.

### 3.9. Schematic of Top Enriched Pathways and Corresponding Gene–Metabolite Association Networks, Highlighting Key Regulatory Molecules in Response to Cold Stress and NCG Intervention in Pairwise Comparisons

**Figure 9:**
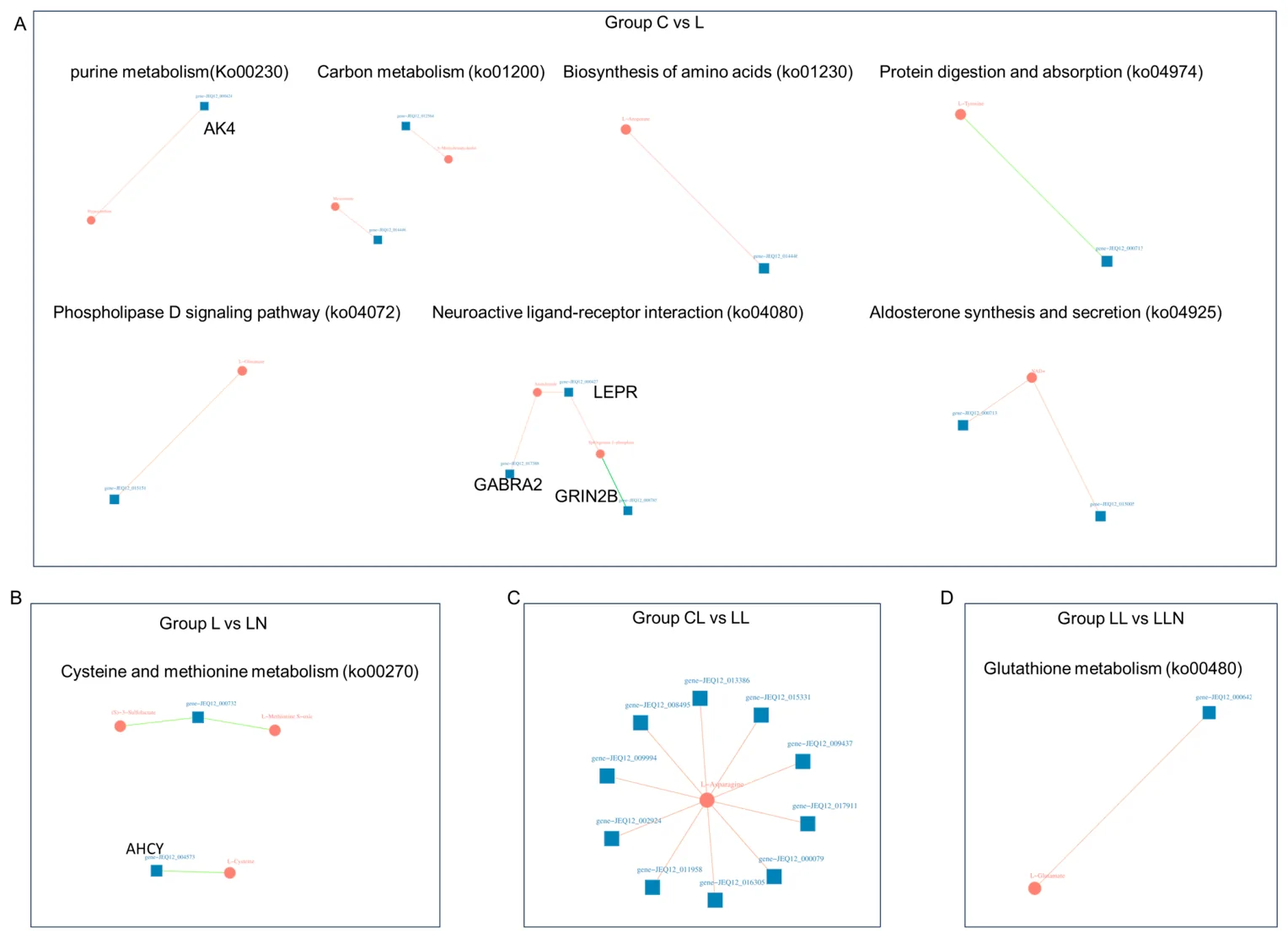
Schematic representation of the top enriched pathways and the corresponding gene–metabolite association networks for pairwise comparisons. (**A**) C vs. L: Key pathways involved in purine metabolism (ko00230), carbon metabolism (ko01200), and phospholipase D signaling pathway (ko04072) were significantly enriched, showing coordinated changes in both transcriptional and metabolic levels. (**B**) L vs. LN: Pathways related to cysteine and methionine metabolism (ko00270), gap junction (ko04540), and neuroactive ligand–receptor interaction (ko04080) were identified as core regulatory modules. The green lines indicate the most significant positive correlations between key genes and metabolites, whereas red lines represent negative correlations. (**C**): Network of DEGs and differential metabolites between Group CL and Group LL. (**D**): Network of DEGs and differential metabolites between Group LL and Group LLN. Red circles represent differential metabolites; blue squares represent DEGs (*AK4* and *RRM2*). The connecting lines indicate significant correlations between genes and metabolites, with different colors representing different association directions.

Integrated transcriptomic and metabolomic analyses were performed to explore the regulatory networks between DEGs and differential metabolites in the liver under different treatments, identifying key pathways and molecular nodes. In Group C vs. Group L (Figure 9A), differential molecules were significantly enriched in multiple pathways, including purine metabolism, carbon metabolism, biosynthesis of amino acids, protein digestion and absorption, phospholipase D signal pathway, neuroactive ligand–receptor interaction, and aldosterone synthesis and secretion. Notably, the AK4 gene was significantly correlated with metabolites in the purine metabolism pathway. In the neuroactive ligand–receptor interaction pathway, key genes such as LEPR, GABRA2, and GRIN2B formed regulatory networks with related metabolites, indicating that these pathways play core roles in the metabolic differences between the two groups. Regarding Group L vs. Group LN (Figure 9B), significant correlations were mainly observed in the cysteine and methionine metabolism pathway, where the AHCY gene was significantly associated with metabolites such as L-cysteine, suggesting that this pathway may be a key regulatory node for amino acid metabolism differences between the two groups. For group CL vs. Group LL (Figure 9C), a star-shaped correlation network centered on the metabolite L-aspartate was formed with multiple DEGs, indicating that L-aspartate metabolism and its related genes are centrally involved in the differential regulation between the two groups and may participate in key metabolic adaptation processes. For group LL vs. Group LLN (Figure 9D), differential correlations were mainly found in the glutathione metabolism pathway, where DEGs were significantly associated with L-glutamate, suggesting that this pathway plays an important role in regulating redox balance between the two groups. In summary, the differential regulation between groups mainly involved key pathways such as energy metabolism (carbon metabolism, purine metabolism), amino acid metabolism (cysteine/methionine metabolism, glutathione metabolism), and signal transduction (neuroactive ligand–receptor interaction). These findings provide key targets and pathway evidence for revealing the molecular mechanism of hepatic metabolic regulation under different treatments.

### 3.10. Differential Gene Expression Validation by qPCR and Transcriptomic Consistency Analysis

**Figure 10:**
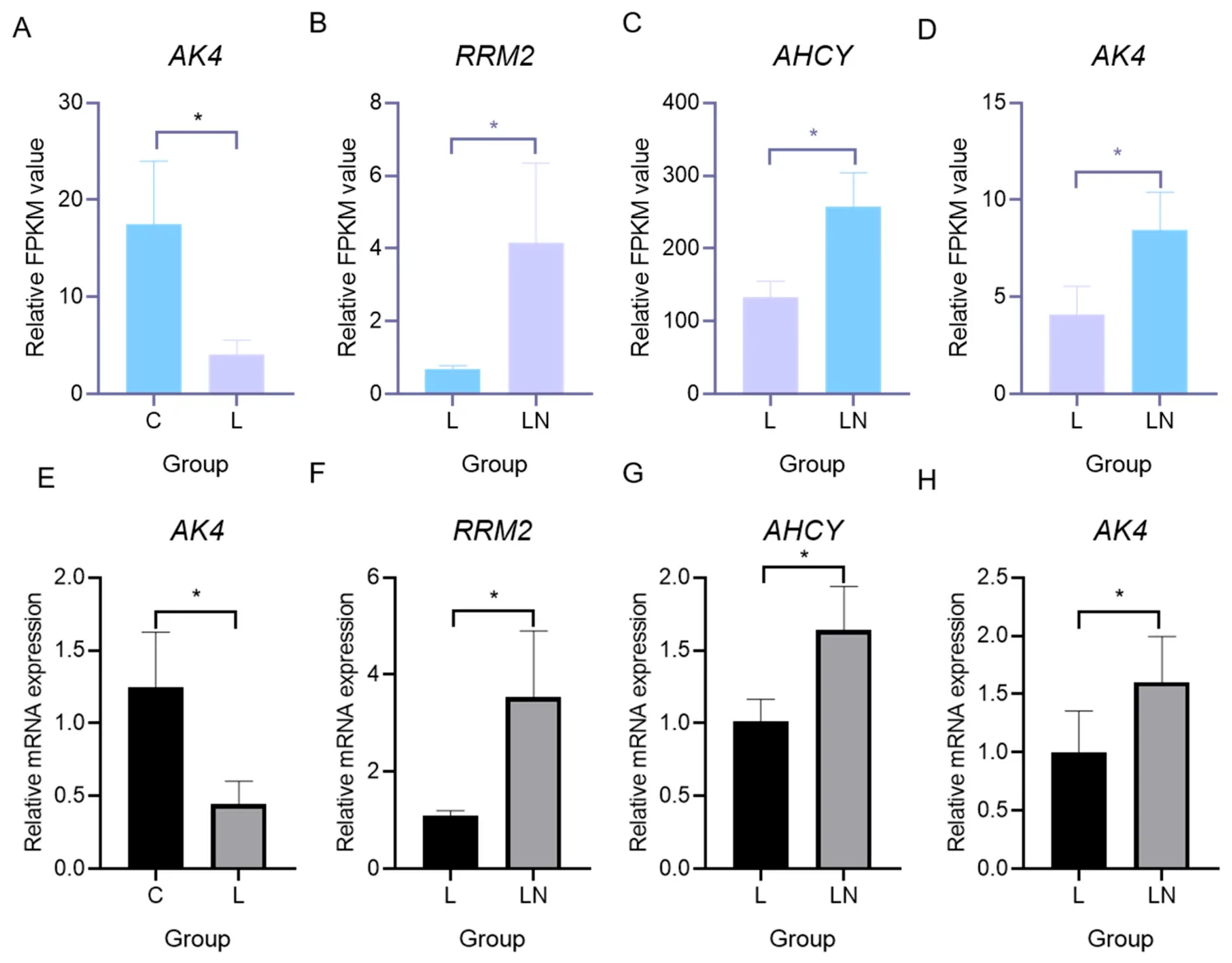
Quantitative validation of key differentially expressed genes (DEGs) via qPCR. (**A**–**D**) FPKM-based transcript abundance of key DEGs (*AK4*, *RRM2*, *AHCY*) in pairwise comparisons RNA-seq results. (**E**–**H**) qPCR verification of the expression patterns of *AK4*, *RRM2*, and *AHCY*. Data are presented as the mean ± standard error of the mean (SEM). Statistically significant differences are denoted by asterisks (*, *p* < 0.05). β-actin (*ACTB*) was used as the internal control. * *p* < 0.05.

This study selected three key differential genes—*AK4*, *RRM2*, and *AHCY*—and employed qPCR technology to verify their expression patterns across different comparison groups (C vs. L, L vs. LN, C vs. LN), correlating these findings with FPKM quantification values. The transcriptomic sequencing results (Figure 10A–D) indicate that, compared to the control group (C), the expression level of *AK4* is significantly downregulated in the cold stress group (L) (*p* < 0.05). In the comparison between the cold-stress and NCG-intervention groups (L vs. LN), both *RRM2* and *AHCY* exhibit significant upregulation (*p* < 0.05), while *AK4* shows a notable trend of recovery. These findings suggest that low-temperature environments substantially disrupt the expression homeostasis of energy metabolism-related genes (*AK4*) and reproductive/developmental genes (*RRM2*, *AHCY*), whereas NCG intervention effectively reverses the expression levels of these genes. The qPCR validation results (Figure 10E–H) align completely with the trends observed in the transcriptomic sequencing, confirming the high reliability of the transcriptomic data from this study. *AK4* (Figure 10E,H): In the cold stress group (L), the expression level is significantly lower than that of the control group (C), while after NCG treatment (LN group), the expression level significantly recovers compared to the L group (*p* < 0.05), indicating that *AK4*-mediated energy metabolism regulation plays a central role in the cold stress response and the improvement mechanisms of NCG. *RRM2* (Figure 10F): A significant expression difference is observed between the L and LN groups (*p* < 0.05), with a notable increase in the LN group, suggesting that *RRM2* may be involved in the cellular repair and metabolic reprogramming processes regulated by NCG. *AHCY* (Figure 10G): As a key gene in folate metabolism and methylation balance, it shows significant expression differences between the L and LN groups (*p* < 0.05), with a substantial increase in the LN group, further confirming that NCG improves cold stress-induced methylation imbalance by regulating the one-carbon unit metabolism mediated by *AHCY*. The qPCR validation results are highly consistent with the transcriptomic sequencing data, not only confirming the accuracy of the differential gene screening in this study but also providing a solid experimental basis for further elucidating the roles of core genes such as *AK4*, *RRM2*, and *AHCY* in cold stress responses and NCG regulatory mechanisms.

## 4. Discussion

This study conducted integrated transcriptomic and metabolomic analyses to demonstrate that NCG supplementation modulated hepatic transcriptional and metabolic profiles, improved metabolic adaptation and energy partitioning, and regulated oxidative stress in cold-exposed ewes and their fetuses, thereby effectively alleviating the adverse impacts of cold stress on the liver of Mongolian ewes. Most previous studies have primarily focused on the effects of cold stress on the intestinal tract (e.g., genome-wide transcriptional regulatory networks in Tibetan sheep intestine [6]), skeletal muscle (e.g., the involvement of calcium signaling, PPAR, and cAMP pathways in thermoregulation [30]), and peripheral tissues. For instance, genome selection for extreme environmental adaptation in Xinjiang Lop sheep has identified cold-tolerant genes such as DNAJC13 and PLCB1 [31]. Nevertheless, integrated transcriptional and metabolic regulation in the liver—the central metabolic organ—under cold stress remains poorly characterized. The present study systematically profiled hepatic molecular regulatory networks in both ewes and fetuses, revealing the differential regulatory effects of NCG between dams and offspring under cold challenge.

As a precursor of arginine, NCG exhibits greater rumen bypass stability and lower cost than direct arginine supplementation, rendering it more suitable for practical ruminant production. Cold stress disrupts the balance between pro-oxidant and antioxidant systems. Under cold exposure, elevated xanthine oxidase activity and decreased catalase activity trigger excessive reactive oxygen species accumulation and intensified lipid peroxidation, ultimately leading to oxidative damage [10]. Endogenous antioxidant enzymes, such as T-SOD and CuZn-SOD, scavenge excess ROS, while MDA serves as a critical biomarker reflecting the degree of in vivo peroxidation and cellular injury [11]. Cold stress also impairs lamb survival rate, animal welfare, and growth performance. Transcriptomic evidence has indicated that immune- and endocrine-related pathways (e.g., TNF signaling pathway and osteoclast differentiation) are activated in lambs exposed to −20 °C, laying a molecular foundation for cold adaptation [32].

Hepatic transcriptomic profiling showed cold exposure induced broad transcriptional changes in energy-metabolism, lipid-synthesis, immune-response and oxidative-stress-related genes. NCG supplementation remodeled the liver transcriptional profile under cold challenge, potentially upregulating mitochondrial function- and ATP-synthesis-related genes and dampening over-activated stress-inflammatory pathways to sustain hepatic metabolic homeostasis and optimize energy allocation for thermoregulation and gestation. These omics observations support a potential regulatory role of NCG in maintaining hepatic metabolic homeostasis under cold stress, which may facilitate rational energy allocation for thermoregulation and gestational maintenance and limit stress-driven inflammatory over-response. Comparison with previous studies revealed conserved as well as species- and stress-type-specific molecular signatures for NCG-mediated stress adaptation. Collectively, our multi-omics data support the hypothesis that NCG acts as a candidate nutritional regulator improving hepatic cold adaptation in pregnant ewes and fetuses. These molecular changes were corroborated by epigenetic regulatory mechanisms underlying ovine metabolic adaptation to cold, including the modulation of SOD1, GPX4, HSP70, and UCP1 [33].

Metabolomic results further showed that cold exposure primarily disrupted amino acid metabolism and energy homeostasis, whereas NCG intervention significantly enriched pathways associated with antioxidant defense, amino acid biosynthesis, and energy metabolism. This indicates that NCG mitigates cold-induced metabolic disturbances by reprogramming core metabolic pathways, providing metabolic-level evidence for its regulatory mechanism. In alpine and hypoxic environments, transcriptomic and metabolomic profiling has been performed on the rumen and heart of Hu sheep. By comparing DEGs linked to rumen microbiota, volatile fatty acids, and digestive metabolism, acetate, propionate, butyrate, and valerate were shown to modulate gene expression. WGCNA analysis demonstrated that distinct digestive metabolic modules are enriched in digestion- and energy metabolism-related pathways and participate in cardiac energy homeostasis. Furthermore, the rumen and gut microbiota play indispensable roles in cold adaptation; cold stress remodels the structure and function of rumen and intestinal microbiota, further regulating host metabolism and health via the gut–heart axis and rumen–meat quality axis [2]. Previous studies have reported that the enrichment of Lachnospiraceae in the rumen increases under cold conditions, and altered propionate metabolism may modulate host immunity without causing severe oxidative damage [5]. In the current study, NCG was found to regulate hepatic oxidative stress and energy metabolism in ewes; however, whether NCG indirectly modulates maternal immune status by reshaping rumen microbial metabolites remains to be further elucidated [3]. Emerging evidence has demonstrated that O-GlcNAcylation participates in metabolic regulation under cold stress in an SIRT1-dependent manner. Ogt knockout exacerbates cold-induced homeostatic imbalance and skeletal muscle mitochondrial oxidative stress, while the Ogt–SIRT axis may contribute to protective effects on skeletal muscle adaptation to cold [4]. These findings imply that NCG may exert systematic anti-stress effects via cross-tissue coordination among the liver, skeletal muscle, and adipose tissue.

Comparisons with other animal models revealed both conserved and unique regulatory patterns. In weaned piglets under heat stress, NCG also reduced MDA content, elevated SOD activity, and activated mTOR signaling. Nevertheless, ewes in this study exhibited milder mTOR activation but stronger antioxidant responses, which may be stress-type dependent: heat stress inhibits mTOR mainly via endoplasmic reticulum stress and inflammation, whereas cold stress induces oxidative damage primarily through ROS burst. Additionally, compared with cold-exposed mice, ovine hepatic gluconeogenic genes (*PCK1*, *G6PC*) showed minimal changes, suggesting that ruminants rely predominantly on rumen-derived VFAs rather than circulating glucose as the major energy substrate under cold stress. Several cold-adaptive candidate genes identified in Xinjiang Lop sheep, such as PLCB1 and HIKESHI [31], overlapped with alterations in the PLC signaling pathway observed in cold-exposed ewe livers, further supporting species-specific adaptive mechanisms. Seasonal shearing and nutritional deprivation in cold seasons trigger adaptive alterations in thermoregulation, antioxidant capacity, and immune function [34,35]. The beneficial effects of NCG observed herein can be regarded as a nutritional boosting strategy that optimizes host adaptation to cold environments. Nevertheless, this study has certain limitations. Firstly, in vitro hepatocyte validation was not performed to distinguish the direct and indirect effects of NCG, nor to clarify whether mTOR activation depends on NCG conversion to arginine or NO production. Secondly, rumen microbiome sequencing was not conducted, leaving unconfirmed whether NCG reshapes rumen short-chain fatty acid profiles (e.g., propionate and butyrate) to further modulate hepatic metabolism in the host [3]. The potential regulatory roles of non-coding RNAs described herein are derived merely from bioinformatic correlation analysis, and these predicted regulatory networks remain to be further verified.

Collectively, this study systematically validated the dual maternal–offspring protective effects of NCG in a ruminant cold stress model. Based on existing literature, the transgenerational-related effects inferred from our liver multi-omics data might be explained by three hypothetical mechanisms: (1) Maternal metabolic reprogramming may supply adequate nutrients for fetal development; (2) NCG may improve placental nutrient transport via arginine-related metabolic pathways; (3) NCG-derived metabolites may pass through the placenta and reshape fetal hepatic metabolic homeostasis. Despite the overall beneficial outcomes, complexity remains: asynchronous changes between hepatic transcriptomic profiles and short-term growth performance suggest that metabolic reprogramming prioritizes gestation maintenance over immediate growth promotion. Moreover, NCG exerted selective modulation on immune indices rather than full restoration, indicating moderate and targeted immune homeostasis regulation to avoid excessive immune activation. In addition, this study focused on late gestation; whether NCG induces long-term developmental programming effects on postnatal lamb growth, immunity, and metabolism warrants further longitudinal investigation. Only the daily feed offer was set, while individual actual feed intake was not monitored. Therefore, we cannot exclude potential differences in voluntary feed consumption induced by cold stress, which should be considered when interpreting the present results. Future research should integrate multi-omics technologies [35] (proteomics, metabolomics, and epigenomics) to identify key molecular targets regulated by NCG. Combined with whole-genome resequencing [36] and GWAS [32], genetic and epigenetic markers associated with cold adaptation in sheep can be further characterized [37]. Production verification under different cold stress intensities is also needed to promote the application of NCG as an eco-friendly and efficient anti-cold stress nutritional regulator in animal husbandry of northern cold regions.

## 5. Conclusions

This study systematically characterized transcriptomic and metabolomic alterations in livers of cold-exposed pregnant Mongolian ewes and fetuses under NCG intervention. Based on multi-omics correlation analysis and qRT-PCR validation, we proposed potential regulatory pathways by which NCG may alleviate cold stress-induced hepatic molecular disorders and modulate maternal and fetal hepatic metabolic profiles. The present findings preliminarily reveal the potential dual protective patterns of NCG in maternal and fetal liver tissues under cold stress. It should be emphasized that all omics-based conclusions in this study are correlative hypotheses rather than functionally verified mechanisms. Further functional experiments, including cell models and pathway validation, will be conducted in follow-up studies to confirm the causal regulatory mechanisms. This work provides valuable candidate genes and metabolic clues for exploring nutritional anti-cold strategies in cold-region sheep farming. NCG reshaped hepatic amino acid and energy metabolic pathways under cold stress at transcriptomic and metabolomic levels. These hepatic molecular alterations reflect the adaptive responses of liver to cold stress and NCG intervention.

## Figures and Tables

**Table 1:** Composition and nutrient levels of experimental diets (air-dry basis)%.

Items	Contents	Nutrient Levels ^②^	Contents
Corn stalk	19.03	DM	89.04
Grain straw	42.51	ME/(MJ/kg)	8.46
Sunflower seed hull	8.46	CP	12.69
Corn	12.52	EE	4.59
Extruded corn	3.53	Ash	8.37
Soybean meal	6.06	NDF	45.26
Cottonseed meal	2.98	ADF	25.73
Distillers Dried Grains with Solubles	2.53	Ca	0.48
Urea	0.46	TP	0.27
CaCO3	0.32	-	
Sodium humate	0.60		
Premix ^①^	1.00		
Total	100.00		

① The premix provided the following per kg diet: Cu 18.00 mg; Mn 40.00 mg; Zn 60.00 mg; I 0.75 mg; Se 0.30 mg; Co 0.30 mg; A 85 000 IU; VD3 25 000 IU; VE 400 mg; CaHPO 4 7 g; NaCl 5 g. ② ME was a calculated value, while the others were measured values.

**Table 2:** Information about primer pairs sequence.

Items	Primer Sequences (5′–3′)	Product Size (bp)	Gene Bank No.
*β-actin*	F: TCAGCAAGCAGGAGTACGAC R: ACGAGGCCAATCTCATCTCG	138	NM_001009784.3
*RRM2*	F: GCCGCTTTGTCATCTTTCCTATCG R: CTTCCCAGTGCTGAATGTCCTTG	120	XM_015094219
*AK4*	F: CCGTGTGCCAGAGGATAGCC R: TGCTTTGCCATATCACCAACTTCC	109	XM_027970883.2
*AHCY*	F: GGGCGAAACGGACGAGGAG	116	XM_004014506.4
	R: TGGTGTGGATGAGGTTGGTGAG		

## Data Availability

The original contributions presented in this study are included in the article. The raw RNA-sequencing data generated in this study have been deposited in the NCBI Sequence Read Archive under BioProject accession PRJNA1252426. Raw untargeted metabolomics data and custom bioinformatics scripts are available from the corresponding author upon reasonable request.

## Supplementary Materials

The following supporting information can be downloaded at: https://www.mdpi.com/article/10.3390/ani16182932/s1, Table S1. Alignment of base quality analysis results in the Mongolian sheep liver. Table S2. Statistical of Sequence Alignment Results Between Sample Sequencing Data and the Selected Reference Genome.

## Abbreviations

N-carbamylglutamate’s (NCG), short-chain fatty acids (SCFAs), malondialdehyde (MDA), total superoxide dismutase (T-SOD), copper-zinc superoxide dismutase (CuZn-SOD), rumen-protected arginine (RP-Arg), real-time quantitative PCR (qPCR), biological process (BP), cellular component (CC), molecular function (MF), FPKM (fragments per kilobase of exon per million fragments mapped), false discovery rate (FDR), Gene ontology (GO), Kyoto encyclopedia of genes and genomes (KEGG), differ-ential expressed genes (DEGs).
