# Supplementary material for: A Study on the Mechanism of N-Carbamylglutamate’s (NCG) Effects on the Liver Transcriptome and Metabolism of Cold-Stressed Mongolian Ewes and Fetuses
Source: Animals (Basel). 2026 Sep 18;16(18):2932. doi: 10.3390/ani16182932 (PMC13603747; doi:10.3390/ani16182932)
Supplement: Supplementary file 1 [file animals-16-02932-s001.zip › animals-4524999-supplementary.pdf]

Supplemental Information

Table S1. Alignment of base quality analysis results in the Mongolian sheep liver.

SampleID	ReadSum	BaseSum	GC(%)	N(%)	Q20(%)	Q30(%)
C1	56,323,212	16,851,088,936	46.31	0	98.54	95.46
C2	55,026,481	16,470,789,736	47.92	0	98.65	95.76
C3	60,771,684	18,171,305,082	47.49	0	98.67	95.73
CL-1	55,408,804	16,589,938,374	47.75	0	98.94	96.65
CL-2	56,403,297	16,885,660,500	47.08	0	98.68	95.80
CL-3	48,364,743	14,481,480,350	47.57	0	98.78	96.02
L1	54,951,243	16,451,048,440	46.52	0	97.86	93.99
L2	49,648,438	14,861,482,914	46.39	0	98.72	95.78
L3	58,089,921	17,387,286,634	47.12	0	98.48	95.25
LL-1	65,753,359	19,676,934,880	47.16	0	97.74	93.80
LL-2	49,996,325	14,959,674,556	47.62	0	98.87	96.52
LL-3	49,099,250	14,699,453,310	47.46	0	98.74	95.96

Notes:

1) SampleID: Sample analysis ID;

2) ReadSum: Total number of pair-end reads in the Clean Data;

3) BaseSum: Total number of bases in the Clean Data;

4) GC(%): GC content of the Clean Data, i.e., the percentage of G and C bases out of the total number of bases in the Clean Data;

5) N(%): Percentage of unresolved bases out of the total number of bases in the Clean Data;

6) Q30(%): The percentage of bases in the Clean Data with a quality score greater than or equal to Q30.

Table S2. Statistical of Sequence Alignment Results Between Sample Sequencing Data and the Selected Reference Genome

BMK-ID	Total Reads	Mapped Reads	Uniq Mapped Reads	Multiple Mapped Reads	Reads Map to '+'	Reads Map to '-'
C1	112,646,424	109,393,690(97.11%)	102,134,637(90.67%)	7,259,053(6.44%)	62,765,206(55.72%)	62,762,707(55.72%)
C2	110,052,962	107,259,915(97.46%)	99,466,354(90.38%)	7,793,561(7.08%)	61,643,675(56.01%)	61,635,831(56.01%)
C3	121,543,368	118,476,271(97.48%)	108,975,433(89.66%)	9,500,838(7.82%)	68,531,406(56.38%)	68,571,454(56.42%)
CL-1	110,006,378	106,428,418(96.75%)	100,830,758(91.66%)	5,597,660(5.09%)	58,453,853(53.14%)	58,406,845(53.09%)
CL-2	110,817,608	108,339,911(97.76%)	102,127,026(92.16%)	6,212,885(5.61%)	60,189,031(54.31%)	60,261,647(54.38%)
CL-3	96,729,486	94,637,772(97.84%)	89,754,672(92.79%)	4,883,100(5.05%)	52,713,847(54.50%)	52,767,742(54.55%)
L1	109,902,486	106,110,772(96.55%)	100,355,120(91.31%)	5,755,652(5.24%)	59,321,490(53.98%)	59,274,676(53.93%)
L2	99,296,876	96,006,514(96.69%)	90,544,263(91.19%)	5,462,251(5.50%)	53,322,991(53.70%)	53,329,894(53.71%)
L3	116,179,842	112,311,843(96.67%)	105,839,048(91.10%)	6,472,795(5.57%)	62,566,984(53.85%)	62,577,355(53.86%)
LL-1	131,506,718	126,816,043(96.43%)	121,075,507(92.07%)	5,740,536(4.37%)	68,836,553(52.34%)	68,867,417(52.37%)
LL-2	99,992,650	94,501,281(94.51%)	91,907,507(91.91%)	2,593,774(2.59%)	49,831,280(49.83%)	49,852,753(49.86%)
LL-3	107,396,786	103,616,903(96.48%)	99,477,330(92.63%)	4,139,573(3.85%)	55,381,633(51.57%)	55,413,461(51.60%)

Note:

1) BMK-ID: BMK's unique sample identifier;
2) Total Reads: Number of clean reads, calculated as single-end reads;
3) Mapped Reads: Number of reads mapped to the reference genome and their percentage of total clean reads;
4) Unique Mapped Reads: The number of reads mapped to a unique location on the reference genome and their percentage of Clean Reads;
5) Multiple Mapped Reads: The number of reads mapped to multiple locations on the reference genome and their percentage of Clean Reads;
6) Reads Map to '+': The number of reads mapped to the positive strand of the reference genome and their percentage of the Clean Reads.
7) Reads Map to '-': The number of reads mapped to the negative strand of the reference genome and their percentage of the Clean Reads.
